# Distribution and Occurrence of Cercospora Leaf Spot of Mungbean (*Vigna radiata*) and Associated Agronomic Factors in Ethiopia

**DOI:** 10.1002/pei3.70194

**Published:** 2026-07-24

**Authors:** Temesgen Bedassa Gudeta, Sandiswa Figlan

**Affiliations:** ^1^ Department of Agriculture and Animal Health, College of Agriculture and Environmental Sciences University of South Africa Florida, Johannesburg South Africa; ^2^ Department of Biology, College of Natural and Computational Sciences Madda Walabu University Bale‐Robe Ethiopia

**Keywords:** cropping system, fungal pathogen, incidence, odds ratio, prevalence, severity

## Abstract

*Mungbean* ( *Vigna radiata* L. Wilczek) is a major cash‐generating pulse crop in Ethiopia's lowlands, serving as a key source of plant‐based protein and contributing to soil fertility. However, its production is severely constrained by Cercospora Leaf Spot (CLS), a destructive fungal disease. During the 2024 and 2025 cropping seasons, a total of 496 mungbean farms from 33 districts in five regions of Ethiopia were inspected to assess the distribution and occurrence of CLS and its association with agronomic factors. The districts were selected according to their mungbean production potential. Necessary information related to farm field inspected including altitude and absolute location were recorded during field assessments. A logistic regression model was used to analyze the associations between CLS occurrence and agronomic factors. CLS was widespread across all surveyed districts, with prevalence ranging from 72% (Gindo Koisha) to 100% (Sodo), and increased from 85.43% of fields in 2024 to 91.25% in 2025, showing a 5.8% increase in CLS occurrence. High CLS incidence (> 50%) and severity (> 40%) were significantly (*p* < 0.0001) associated with Sodo districts, followed by Adami Tullu, Awash Melakassa, Kulfo‐Halaba, and Lebo Kemkem. These conditions were predominantly observed in the 2025 cropping season at elevations above 1500 m a.s.l. and were linked to high weed infestation, minimal tillage, and infrequent weeding during the grain‐filling stage. Therefore, priority should be given to CLS‐prone districts through research‐based integrated management to ensure sustainable mungbean production. This would enhance nutritional security and recognize mungbean as a future smart food crop.

## 1 Introduction

Mungbean ( *Vigna radiata* L. Wilczek) is a self‐pollinated diploid legume crop belonging to the family Fabaceae and characterized by alternate trifoliate compound leaves (Lema et al. 2018; Teferie et al. 2020; Gudeta et al. 2024). It plays an important role in nutritional security, sustainable farming systems, and crop rotation due to its nitrogen‐fixing ability. Mungbean grows quickly even under temperatures above 20°C, with a growth cycle of about 2 months from planting to harvest (Batzer et al. 2022). It is a drought‐tolerant, warm‐season pulse crop cultivated worldwide primarily for its protein‐rich seeds, sprouts and forage (Nair et al. 2019; Das et al. 2019; Kim et al. 2025). High‐quality protein from whole and processed mungbean seeds is used as sprouts, and in soups, bean paste, ice cream, noodles, and desserts (Rizvi et al. 2022; Kim et al. 2025). It has wide adaptability (Irfan et al. 2025), a range of cropping systems (Teferie et al. 2020; Kessy et al. 2024), low input requirements and the ability to improve the soil by fixing atmospheric nitrogen (Gudeta et al. 2024; Pattnaik et al. 2024).

In Ethiopia, mungbean is integrated into a double‐cropping system with cereal crops, to enhance land use efficiency. Cultivation mainly occurs during the short rainy season in warm agro‐ecologies (Bejiga and Teressa 2021; Dikr 2023; Idris et al. 2025). Due to its adaptability to low rainfall conditions, farmers in drought‐prone areas grow it to supplement their protein needs and generate income (Teferie et al. 2020; Gata et al. 2024). For the last few decades, mungbean in Ethiopia, has become a crucial component of cereal‐based cropping systems, contributing significantly to food security, income for smallholder farmers and soil health (Mota et al. 2021; Dikr 2023; Kebede and Kasim 2024). Consequently, mungbean is now being cultivated on a large scale for commercial purposes (Kassa et al. 2022; Yahya and Teshome 2024; Idris et al. 2025). Despite its importance for nutrition, feed, and soil fertility, mungbean production in Ethiopia faces several challenges that limit yield stability and grain quality. Although mungbean production is currently expanding rapidly in Ethiopia, several studies indicate that its productivity and average yield have lagged behind those of Asian countries (Ademe 2023; Tehulie et al. 2021; Dinsa et al. 2022; Dikr 2023). Evidence from the national central statistical agency (CSA 2022), value‐chain analyses (Assefa et al. 2022), and agronomic studies (Tehulie et al. 2021; Baza et al. 2022) show that the cultivated area and number of mungbean producers are rising quickly in Ethiopia, mainly driven by export demand and drought resilience. However, the productivity and grain quality of mungbean still lag behind due to soil fertility problems, lack of improved varieties, pests and diseases, abiotic stresses, and weak input and market systems (Kassa et al. 2022; Baza et al. 2022; Dikr 2023). Studies from abroad also indicate that biotic stresses such as insect pests (e.g., bruchids, whitefly, thrips, aphids, pod borers) and diseases (yellow mosaic, anthracnose, powdery mildew, Cercospora leaf spot, bacterial and halo blights) substantially depress productivity and marketable quality (Nair et al. 2019; Van Haeften et al. 2023; Rashidi et al. 2025). Abiotic stresses such as drought, heat, salinity, and waterlogging critically limit growth, reproductive success, and seed quality, especially under climate change. Moreover, poor agronomic practices, lack of improved and stress‐tolerant varieties, and shortage of quality seed worsen these challenges, resulting in global yields far below the crop's potential (Chankaew et al. 2021; Dikr 2023). Under Ethiopian conditions, mungbean production and quality are significantly constrained by several biotic stresses such as foliar diseases. Among the foliar diseases, fungal diseases such as CLS, caused by the fungal pathogen *Cercospora canescens* Ellis & G. Martin (syn. *C. cruenta* ), is one of the most widespread and economically destructive foliar diseases of mungbean worldwide (Shahzady et al. 2017; Chauhan et al. 2022; Gudeta et al. 2024; Mota et al. 2021). The symptoms of CLS are characterized by circular to irregular reddish‐brown to dark brown spots on leaves, often surrounded by chlorotic haloes. Under severe infection, these lesions coalesce, leading to premature leaf shedding, reduced photosynthetic area, poor grain fill, and substantial yield losses, which can range from 20% to 80% depending on environmental conditions and the cultivar's susceptibility (Sharma et al. 2021). Globally, CLS is endemic and frequently epidemic in major mungbean‐producing countries such as India, Pakistan, Bangladesh, Thailand, the Philippines, China, and Australia (Prasad et al. 2021; Gudeta et al. 2024; Yadav et al. 2024). The pathogen is seed‐borne and can also survive on infected crop residues, facilitating its long‐distance dissemination and persistence in agro‐ecosystems (Chauhan et al. 2022).

The occurrence and severity of CLS are not uniform but are intricately linked to a number of agronomical and climatic factors. Climatic factors such as warm temperatures (25°C–30°C), high relative humidity (> 80%), and prolonged leaf wetness from frequent rainfall or overhead irrigation are the primary drivers of CLS epidemics (Bhardwaj et al. 2023). These conditions support the pathogen's conidial production, germination, and penetration of the host epidermis (Pal et al. 2017). Poorly drained heavy soils that retain moisture can elevate plant canopy humidity (Miah et al. 2019). Besides, nutrient imbalances, particularly excessive nitrogen, potassium, and phosphorus deficiencies, have been associated with increased disease severity (Mota et al. 2021). Cropping system and farming practices including monocropping of mungbean or rotation with other susceptible legume hosts lead to inoculum buildup for CLS. Close plant spacing reduces air circulation, prolonging canopy wetness. Late sowing often aligns the crop's susceptible growth stages with peak periods of favorable weather (Neupane et al. 2023). The limited use of improved resistant cultivars in many regions exacerbates the problem (Belay et al. 2019). Understanding the distribution and occurrence of CLS across the mungbean‐growing areas of Ethiopia and the dynamic interactions between the disease and agro‐ecological factors is fundamental for developing integrated disease management strategies for CLS. Research on the spatial and temporal patterns of CLS in relation to these agronomic factors provides critical insights for predictive modeling, risk assessment, and targeted management interventions to secure mungbean production. Agronomic factors are the components and practices of crop production that influence yield, quality, and overall sustainability, including soil type, water availability, nutrient management, and crop management techniques (Petersen et al. 2023). However, knowledge of the current status and mungbean management practices particularly for CLS in Ethiopia remains limited. Additionally, the influence of various agronomic factors and their relationship with CLS occurrence remains poorly understood. Consequently, mungbean production and productivity in many parts of the country have declined due to the damaging effects of CLS (Mota et al. 2021; Dikr 2023; Kebede and Kasim 2024). Hence, for systematic disease monitoring, it is necessary to obtain empirical data on the distribution, type, and relative significance of CLS, as well as to evaluate the role of agronomic factors in CLS development. To address this, the present field survey was conducted to examine the geographic distribution of CLS in mungbean and its association with agronomic factors in Ethiopia.

## 2 Materials and Methods

### 2.1 Description of the Study Areas

**FIGURE 1:**
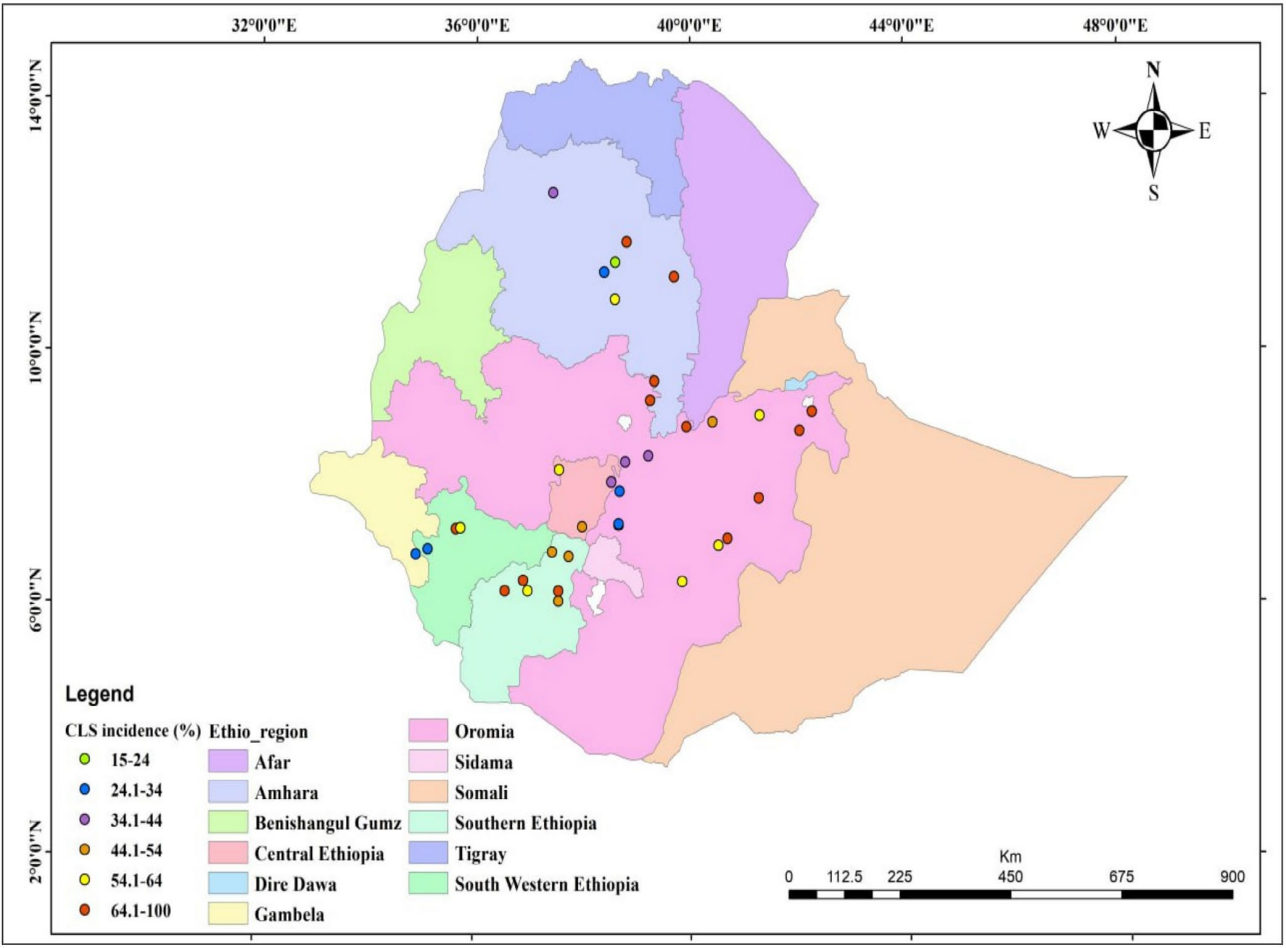
CLS Incidence (%) across the major mungbean‐growing districts of Ethiopian regions.

Mungbean farm fields were surveyed during the 2024 and 2025 main cropping seasons across 33 purposively selected major mungbean‐growing districts in five national regions of Ethiopia, indicated in Figure 1 and Table S1. The survey areas were identified based on information from the Melkassa Agricultural Research Centre (MARC), the national center of excellence for mungbean and other lowland pulse research. The number of districts within each region was determined based on the production potential and the extent of area dedicated to mungbean production and their exposure to CLS. The data on climatic conditions in the study districts during the 2024 and 2025 cropping seasons, including mean rainfall, temperature, and relative humidity, were obtained from National Meteorological Agency branches and nearby local stations (Table S1). The altitudinal elevation and geographic locations of each mungbean farm were recorded using a Garminetrex GPS (Global positioning system) device during the field survey.

### 2.2 Procedure of Sampling and Sampling Unit Determination

The sampling units (mungbean farm fields) within each district were selected randomly along accessible roads at 5–10 km intervals from an initial reference field. CLS disease incidence and severity were documented using a 1 m × 1 m quadrat at five spots (observations points) in each mungbean field by moving in an X‐fashion transect from the initial point. Each of the corners of the four quadrants was 10 m apart from one another in the sampled field. Considering that mungbean cropping seasons differ by locality in Ethiopia, especially in bimodal rainfall areas where farm field numbers vary between seasons, the survey was carried out in collaboration with local agricultural offices in each study district. To ensure accuracy, two agricultural experts per district were trained and were assigned to monitor field conditions, collect data and report observations during the two study seasons. Over the two study years (2024 and 2025) for the CLS assessment, the mungbean farm fields were inspected from the flowering to maturity stages of the crop growth cycle, following the localities and the cropping seasons. This was done because the onset of CLS on the mungbean farm most commonly occurs at flowering, with major outbreaks at pod setting and grain filling, progressing to maturity (Sahoo et al. 2022; Das et al. 2019). The same 33 study districts surveyed during the 2024 main cropping season were repeated in 2025 using the same sampling and disease assessment procedures. Because the study design was a farmer field–based cross‐sectional survey, the same individual mungbean field could not always be reassessed in the subsequent season (during 2025), as some farmers frequently rotated crops or planted mungbean in different fields. In such cases, another representative mungbean field within the same district was selected and inspected following the same standardized sampling and disease assessment protocols. Standardization of sampling was used to minimize confounding due to plant age, in which data collection time was standardized to the flowering stage (early pod‐setting), about 40–50 days after sowing, across all districts and years. This ensures that all percentage disease incidence (PDI) and percentage severity index (PSI) values are comparable across districts over the two seasons. Then pooled data across two cropping seasons (2024 and 2025) was used to reduce the bias of a single‐year snapshot. A total of 496 mungbean fields were assessed across 33 districts during the study period. Four kebeles were purposively selected per district. Within each kebele, 3–5 fields were assessed based on availability and farm size, resulting in 12–20 fields per district. Libo Kemkem district was an exception, where only eight fields were assessed (Table 2).

### 2.3 Mungbean Field Inspection Procedure for CLS Symptom Identification

During field inspection, a detailed and systematic examination of CLS symptoms was carried out during pod setting and grain filling, as these are the growth stages of the mungbean plant when CLS typically appears and intensifies in the field. In addition to the X‐shaped transect quadrant used for field evaluation, a W‐pattern walk was conducted across the field to enhance the detection of CLS infected leaves. Twenty plants per quadrat were randomly sampled, and both upper and lower leaves were examined for CLS symptoms. At the initial stage of the study, particular attention was focused on the older, lower leaves at the base of the plant. This was because lower leaves lie closer to the soil and are therefore more likely to be infected early, as plant debris in the soil is the primary source of the CLS pathogen inoculum. At the early stage CLS diagnosis, the appearance of very small necrotic (dead) and water‐soaked gray to brown central spots bordered by the leaf veins was clearly observed on the leaves. A hand lens was used to more closely examine the dense, unclear spots on the leaves. By undergoing weekly continuous follow‐up on the sampled plants, the spots on the infected leaves were observed as expanded, irregular to angular‐shaped lesions with typical gray spots having dark‐reddish margins and yellow halos. In some of the assessed fields, premature defoliation and reduced pod numbers were also observed as the CLS disease progressed to maturity across the seasons. To maintain specificity in CLS severity and incidence estimates, we applied a strict inclusion criterion: only fields where CLS was the dominant (> 80% of total symptomatic leaf area) were retained for quantitative PDI (%) and PSI (%) estimations. Fields with heavy mixed infections, ambiguous symptoms, or where other pathogens contributed > 20% of foliar damage were excluded from the final dataset. This approach minimized the potential confounding effects of co‐infections and ensured that the epidemiological indices accurately reflected CLS distribution.

### 2.4 CLS Disease Assessments

Based on recurrent reports from farmers and agricultural experts assigned to each study district, CLS disease assessments were conducted during the study period. Mungbean farmers were informed in advance by district agricultural experts to regularly monitor their fields and immediately report any foliar disease symptoms. Trained agricultural experts subsequently identified and assessed CLS symptoms. The CLS‐infected and uninfected mungbean plants within the quadrant were scored per field. Then, the prevalence of CLS across the surveyed districts in each study year was estimated using the following formula (Yadav et al. 2024).

(1) $$\mathrm{CLS}\;\text{prevalence}\%=\frac{\text{Number of}\;\mathrm{CLS}\;\text{diseased mungbean farm fields}}{\text{Total number of mungbean farm fields assessed}}\times 100;$$

Plant population in each quadrat was counted, considering individual plants as sampling units. The mean plant population density was obtained by averaging the mungbean plant population in the five quadrants of each field. The average incidence and severity values of CLS were estimated from each of the five quadrants (observations) per field for data analysis. The CLS disease severity rating scale was evaluated using a 0–9 scale used by Das et al. 2019, where 0 = no visible symptom of disease; 1 = 0.1%–10% foliage affected; 2 = 10.1%–20% foliage affected; 3 = 20.1%–30% foliage affected; 4 = 30.1%–40% foliage affected; 5 = 40.1%–50% foliage affected; 6 = 50.1%–60% foliage affected; 7 = 60.1%–70% foliage affected; 8 = 70.1%–80% foliage affected; and 9 = > 80% foliage affected. The CLS severity scores for each plant assessed in each field were added and divided by 20 (the total number of mungbean plants randomly assessed per quadrant) to arrive at the mean severity score per field. The CLS severity scores were subsequently converted to PSI for analysis. Both PSI and PDI were calculated using the following formulae (Rehman et al. 2021; Chand et al. 2025):

(2) $$\mathrm{PSI}\;\left(\%\right)=\frac{\sum \left(\text{numerical ratings}\right)}{\text{Number of plants scored}\times \text{maximum score}\;\mathrm{on}\;\text{scale}}\times 100;$$

(3) $$\mathrm{PDI}\;\left(\%\right)=\frac{\text{Number of diseased mungbean plants in the quadrant}\;}{\text{Total number of mungbean plants in the quadrant}}\times 100;$$

Moreover, data on associated agronomic factors such as cropping system, sowing patterns, farm‐field size, seed source, tillage frequency, sowing time, soil type, plant density, growth stage (vegetative, flowering, and grain filling), weeding frequency, level of weed infestation, inorganic fertilizer application and presence or absence of waterlogging were gathered by assessing the mungbean farm fields. In the present study, cropping systems were either sole or mixed cropping, with sole cropping referring to mungbean grown in monoculture. Mixed cropping refers to mungbean grown with other crops, such as maize, finger millet, sorghum, potato, enset, and coffee plants, depending on the localities. The sowing pattern of mungbean cultivars across the study districts was also assessed by visual field observation, whether grown by broadcasting or row planting. Status of weed infestation in each mungbean field was qualitatively assessed and recorded on an ordinal scale: “none” for totally weed‐free fields; “low” for slight or trace weeds covering less than a quarter of the field area, “moderate” for fields with non‐dense weeds covering about half of the field area, and “severe” for fields with heavy and densely overgrown weeds. During field inspection, the soil types in each sampled farm field were identified based on landscape features, color and the extent of cracks (when dry) by examining the surface of the farmland. Accordingly, two soil types were recognized across the sampled mungbean fields as Nitisol and Vertisol. Accordingly, when the soil type was observed as red‐dark or reddish‐brown in color with no or minor cracks across the mungbean fields on rolling or undulating sloped landscapes, the soil type was recorded as Nitisol. The soil type was recognized as Vertisol, when the field's landscape was observed as flat depressions with the black or dark‐gray topsoil having deep, wide open cracks (Elias 2017; Mathewos and Mesfin 2024; Teshale 2023; Tlili et al. 2025). When the surface of the farmland was observed to have deep, wide cracks when dry, it was recorded as clay soil. Other necessary information on the other key agronomic variables, such as CLS management practices, CLS disease awareness, weeding regularity, farm size, seed sources, and constraints (lack of resistant seeds, lack of inorganic fertilizer, no fungicide use, waterlogging), was collected via a structured questionnaire for mungbean farmers. Data on farm‐land tillage before sowing, time of sowing, mungbean cultivars used, weeding practice, fertilizer and fungicide application, and waterlogging were gathered through direct interviews with farmers during the field survey.

### 2.5 Data Analysis

The collected data were analyzed using IBM SPSS statistics for windows, Version 29.0 (IBM Corp 2023). Descriptive statistical analysis was performed to examine the prevalence, distribution and association between CLS disease incidence and severity, and agronomic factors across administrative districts. To address this, data of agronomic factors and that of disease intensity (DI% and PDI% of CLS) were first recorded at the individual mungbean field level (each inspected field was used as the observational unit) in each district and then statistically analyzed. Following the field‐level analyses, district‐level mean values were estimated by averaging the data observations from all surveyed fields within each district. These district means data were used only for descriptive presentation in tables to facilitate comparison among districts and were not used as the primary unit for statistical inference. This provided the variability among the study districts. During the analysis, the susceptibility of mungbean plants to CLS in the inspected fields was considered above the estimated means for both incidence (> 50%) and severity (> 40%), which were also used as class boundaries. The assessed CLS incidence (DI%) and severity (PSI) were dichotomised (categorized) into binomial qualitative groups (boundary cut point) as ≤ 50% and > 50% for incidence, and ≤ 40% and > 40% for severity (Debele and Ayalew 2015; Nayak et al. 2018). Based on these cut points of data boundaries, the bivariate distribution of both incidence and severity of CLS was signified by generating a cross‐tabulation along with that of the agronomic factors (Table 1). Therefore, a standard binary logistic regression statistical model was used to analyze the association of each of the two dependent variables with the predictor variables (Wato et al. 2021; Tang et al. 2023) using SPSS version 29. To identify the most significant agronomic factors influencing CLS incidence and severity, a reduced logistic regression model was used. To determine the association between each agronomic predictor and the dichotomised responses for CLS incidence and severity, a univariate logistic regression model within a generalized linear model (GLM) was applied with the log odds model of the outcome as a linear function of each predictor (Tang et al. 2023).

(4) $$\text{Logit}\;\left(p\right)=\ln\left(\frac{p}{1-p}\right)=\beta_{0}+\beta_{1}X_{1}+\beta_{2}X_{2}+\cdots \beta_{p}X_{p};$$

where *p* represents the probability that the CLS incidence occurs, considering the specific values of the agronomic (independent) variables; $\frac{p}{1-p}$ and $\ln\left(\frac{p}{1-p}\right)$ represent odds and the natural log of odds, respectively. *β* is a constant agronomic coefficient that represents the change in the log odds of the disease occurring for a one‐unit change in the agronomic predictor variable, holding all other variables constant; *X* is the agronomic variable. This enabled the analysis to depend only on the most important predictive and explanatory agronomic factors. Moreover, a reduced multivariate logistic regression model was applied to further assess the significant associations between independent variables and the disease incidence and severity by estimating the odds ratios and deviance reduction for each variable (Yuen 2006; Vakhitova and Alston‐Knox 2018).

(5) $$\text{Deviance}\;\left(∆D\right)=-2\sum_{i=1}^{n}\left[\left[y_{i}\ln\left(p_{i}\right)\right]+\left(1-y_{i}\right)\ln\left(1-p_{i}\right)\right];$$

*p*_*i*_ predicted probability for the *i*th observation (farm field); *y*_*i*_ is the actual observation of CLS occurrence in terms of incidence or severity.

**TABLE 1:** Agronomic factors and their variable categories in the occurrence rate of CLS across the inspected fields of mungbean‐growing districts of Ethiopia during the 2024 and 2025 cropping seasons.

Variables	Variable category	Number of fields	Prevalence (%)	Incidence		Severity		PDI (%)	PSI (%)
				> 50%	≤ 50%	> 40%	≤ 40%	Mean ± SE	Mean ± SE
Year	2024	199	83.89	96	103	76	123	47.8 ± 2.3	38.2 ± 3.3
	2025	297	87.56	154	143	129	168	52.3 ± 1.3	42.1 ± 0.5
Altitude	≤ 1000	84	73.30	34	50	24	60	38.6 ± 4.0	28.4 ± 5.3
	1000–1500	341	88.53	174	167	133	208	50.1 ± 1.2	39.0 ± 0.4
	≥ 1500	71	94.42	44	27	36	35	61.2 ± 7.5	50.4 ± 4.7
Cropping system	Sole cropping	394	88.53	207	187	158	236	52.1 ± 1.7	40.0 ± 1.5
	Mixed cropping	57	96.67	35	22	30	27	61.7 ± 6.2	52.6 ± 3.7
	Intercropping	45	72.46	14	31	9	36	31.5 ± 5.3	19.5 ± 5.0
Sowing pattern	Row sowing	203	84.22	71	132	47	156	50.7 ± 4.7	41.2 ± 3.7
	Broadcasting	293	87.03	205	88	194	99	53.5 ± 3.2	43.1 ± 0.6
Soil type	Vertisol	294	84.16	129	165	102	192	48.4 ± 6.4	39.7 ± 4.0
	Nitisol	202	87.35	115	87	95	107	51.7 ± 4.1	41.0 ± 2.1
Plant density	≤ 40 m^−2^	190	79.72	78	112	53	137	40.6 ± 6.4	27.7 ± 4.4
	> 40 m^−2^	306	91.43	190	116	161	145	61.4 ± 1.1	52.4 ± 0.6
Plant growth stage	Vegetative	35	67.67	11	24	7	28	31.4 ± 3.0	18.0 ± 2.6
	Flowering	149	90.36	79	70	69	80	52.7 ± 1.7	44.5 ± 5.1
	Grain‐filling	312	98.67	206	106	184	128	65.5 ± 0.5	58.7 ± 1.0
Weed infestation level/status	None (clean)	64	66.71	21	43	16	48	32.7 ± 5.0	24.0 ± 0.7
	Low	262	84.72	121	141	94	168	45.4 ± 0.3	35.3 ± 1.1
	Moderate	143	92.83	83	60	70	73	57.2 ± 3.3	48.4 ± 2.6
	High	27	97.32	17	10	15	12	64.3 ± 5.5	56.1 ± 5.3
Weeding frequency	Once	93	97.21	70	23	62	31	73.7 ± 1.5	66.0 ± 6.7
	Twice	371	89.72	145	226	111	260	38.1 ± 0.3	29.5 ± 1.2
	≥ 3 times	32	69.54	12	20	8	24	35.7 ± 6.7	24.3 ± 5.0
Tillage (plowing practice)	2 times	261	94.06	162	99	136	125	62.7 ± 5.2	48.0 ± 3.0
	3 times	148	87.53	68	80	52	96	47.5 ± 0.5	38.5 ± 0.7
	4 times	87	74.67	37	50	27	60	38.7 ± 4.5	31.5 ± 5.5
Field size	≤ 0.25 ha	351	85.33	179	172	140	211	49.4 ± 5.7	38.2 ± 6.7
	> 0.25 ha	145	87.12	75	70	61	84	52.5 ± 6.3	41.1 ± 1.3
Mungbean cultivar used	Improved	147	80.27	53	94	35	112	39.5 ± 1.2	28.1 ± 0.7
	Traditional	349	91.40	224	125	186	163	60.3 ± 7.0	48.2 ± 1.5
Names of CI varieties	Rasa (N‐26)	61	85.56	92	112	75	129	44.1 ± 0.6	35.2 ± 0.4
	NVL‐1	58	72.52	73	124	53	144	37.0 ± 1.5	26.7 ± 3.5
	Shewarobit	28	93.56	66	29	57	38	68.3 ± 4.2	59.0 ± 6.1
Seed Source	Agri‐office	56	72.34	19	37	15	41	33.2 ± 1.2	24.7 ± 5.4
	Local market	299	95.14	209	90	188	111	69.6 ± 0.4	62.7 ± 0.5
	Farmer	141	88.67	67	74	54	87	47.3 ± 6.0	37.7 ± 4.0
Sowing time	Mid‐February	115	79.54	52	63	39	76	42.5 ± 3.4	31.3 ± 6.7
	Early April	28	85.67	13	15	11	17	48.7 ± 4.0	38.0 ± 6.2
	Mid‐July	353	92.46	222	131	194	159	60.7 ± 1.1	52.2 ± 0.6
Inorganic fertilizer	Applied	468	75.45	164	304	122	346	35.4 ± 7.7	25.3 ± 2.0
	Not applied	28	95.70	18	10	15	13	63.1 ± 0.6	54.2 ± 0.5
Fungicide application	Sprayed	25	84.68	12	14	10	16	46.7 ± 3.3	37.3 ± 2.1
	Unsprayed	471	86.57	259	212	198	273	50.5 ± 6.7	40.4 ± 5.7
Waterlogging	Present	259	96.33	99	18	93	24	65.4 ± 2.0	51.1 ± 1.3
	Absent	237	74.07	241	138	234	145	47.1 ± 0.6	38.4 ± 1.2

*Note:* Variables = Agronomic factors; > 50% and ≤ 50% cut points of highest and lowest CLS incidence intervals, whereas > 40% and ≤ 40% are for cut points of highest and lowest CLS severity intervals, respectively.

Abbreviations: CI varieties, cultivated improved varieties; SE, standard error.

For CLS severity:

(6) $$\text{Logit}\;\left(P\right)=\ln\left(\frac{P\left(Y\leq j\right)}{P\left(Y>j\right)}\right)=\alpha_{j}-\left(\beta_{1}X_{1}+\beta_{2}X_{2}+\cdots \beta_{p}X_{p}\right);$$

where *α*_*j*_ represents the intercept (threshold) values for each CLS severity level *j*. The agronomic coefficients *β* are constant across CLS severity levels.

In the present analysis, deviance reduction was used as a measure of model fit improvement based on the significant variables enabling the model itself to examine the causal variables that affected the occurrence and severity of CLS. Odds ratios were used to estimate the strength and direction of the relationship between each explanatory variable and the binary outcome, making them useful for understanding how specific factors influence the likelihood of CLS. The likelihood ratio tests against *F*‐statistics and *χ* ^2^ (Chi‐squared) values were used to test the significance of the independent variables (Tang et al. 2023; Nayak et al. 2018). Variables with statistically significant effects at (*p* < 0.0001) were used for interpretation. To interpret log odds and get the odds ratio (OR), *β* was exponentiated as

(7) $$\text{Odds ratio}=e^{\beta }\;\text{elaborated}\;\mathrm{as}:e^{\beta 0+\beta_{1}X_{1}+\beta_{2}X_{2}+\cdots \beta_{p}X_{p}};$$

To get the actual predicted probability (*P*) of CLS disease from the reduced model the inverse logistic function was used following (Wang and O'Connor 2019) as follows:

(8) $$P=\left(\frac{e^{\beta 0+\beta_{1}X_{1}+\beta_{2}X_{2}+\cdots \beta_{p}X_{p}}}{1+e^{\beta 0+\beta_{1}X_{1}+\beta_{2}X_{2}+\cdots \beta_{p}X_{p}}}\right);$$

In the present study, the value of deviance reduction (*D*↓) greater than that of the residual deviance (the error of the fitted model after predictors were added) which is relatively nearer to the value of the intercept or the null deviance (error of a model with no predictors or before the factors are added) indicates the improvement or the better fit of the model used. When the estimated value of deviance reduction (*D*↓) is greater than the residual deviance but closer to the value of the intercept or the null deviance, then the model best fits the analysis (Di Mari et al. 2023). A larger *D*↓ is interpreted as a higher *χ* ^2^ value and a smaller *p*‐value, indicating that the model fits enough to predict the outcome variables based on the entered independent (predictor) variable (Ross 2017; Di Mari et al. 2023). In this logistic regression analysis, the likelihood ratio test was used to determine variable significance. For each categorical predictor, the category with an odds ratio (OR) of 1.00 was designated as the reference group. This choice establishes the baseline against which all other categories are compared and does not influence the overall model fit or the statistical significance of the variable. The district that had OR = 1 with a relatively large sample size and less exposure to CLS was designated as the reference district to confirm smaller standard errors (SE) for the estimated coefficients, resulting in more stable model estimates (ME) (Norton and Dowd 2018; Vsevolozhskaya et al. 2018; Di Mari et al. 2023).

## 3 Results

### 3.1 Characteristics of Surveyed Mungbean Fields and CLS Prevalence

**FIGURE 2:**
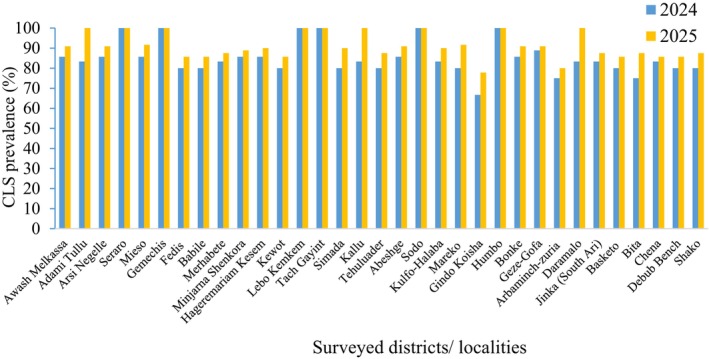
Prevalence (%) of CLS in potential mungbean‐growing districts of Ethiopia, during the 2024 and 2025 cropping seasons.

In the present study, the occurrence and distribution of CLS was assessed along with associated agronomic factors across 33 major mungbean‐growing districts in 17 zones of five Ethiopian regional states (Table S1; Table 2). CLS was found to be widely distributed across all surveyed districts with a variable prevalence rate. Among the 496 mungbean fields surveyed across the 2024 and 2025 cropping seasons, 441 (88.9%) exhibited CLS symptoms, while only 55 (11.1%) were disease‐free. Of the total fields assessed, 199 (40.1%) were surveyed in 2024 and 297 (59.9%) in 2025, all from the same major mungbean‐growing districts targeted during 2024. CLS was detected in 170 (85.43%) fields in 2024 and 271 (91.25%) fields in 2025, representing a 5.8 percentage‐point increase in CLS disease occurrence between the two survey years. These findings indicate that CLS was highly prevalent across Ethiopia's major mungbean‐growing areas and became more widespread in 2025, reflecting a year‐over‐year increase in CLS disease pressure (Figure 2).

**TABLE 2:** Mean percent incidence (PDI) and severity index (PSI) of CLS in major mungbean‐growing districts of Ethiopia during 2024 and 2025 cropping seasons.

Region^a^	Zone	Districts/Localities	No. of field^b^	Incidence		Severity		PDI (%)	PSI (%)
				> 50%	< 50%	> 40%	< 40%	Mean ± SE	Mean ± SE
Oromia	East Shewa	Awash Melkassa	18	12	6	11	7	68.3 ± 7.2	61.1 ± 6.0
		Adami Tullu	17	12	5	11	6	70.7 ± 5.5	64.5 ± 4.5
	West Arsi	Arsi Negelle	18	9	9	7	11	51.6 ± 8.0	40.5 ± 5.7
		Seraro	16	9	7	8	8	55.2 ± 3.0	42.3 ± 4.2
	West Harargae	Mieso	19	5	14	4	15	28.6 ± 1.1	17.3 ± 1.4
		Gemechis	15	9	6	8	7	59.3 ± 4.3	46.4 ± 1.3
	East Harargae	Fedis	12	4	8	3	9	33.6 ± 1.4	25.7 ± 2.6
		Babile	12	6	6	5	7	49.3 ± 1.3	41.0 ± 2.7
Amhara	North Shewa	Merhabete	14	8	6	6	8	54.0 ± 7.0	44.3 ± 6.0
		Minjarna Shenkora	16	8	8	7	9	50.4 ± 6.2	40.4 ± 3.7
		Hageremariam Kesem	17	7	10	6	11	42.8 ± 2.2	34.4 ± 4.5
		Kewot	12	6	6	5	7	52.0 ± 6.0	44.2 ± 2.7
	South Gondar	Lebo Kemkem	8	5	3	5	3	65.5 ± 4.7	55.5 ± 2.0
		Tach Gayint	14	8	6	8	6	59.4 ± 5.5	51.7 ± 4.5
		Simada	15	8	7	7	8	52.9 ± 1.7	43.0 ± 5.0
	South Wello	Kallu	14	9	5	9	5	61.1 ± 8.1	53.7 ± 5.0
		Tehuluader	13	4	9	3	10	30.7 ± 7.4	20.8 ± 2.0
Central Ethiopia	Guragae	Abeshge	18	10	8	8	10	52.1 ± 7.1	41.0 ± 2.7
		Sodo	15	12	3	10	5	72.4 ± 3.4	67.0 ± 2.2
	Halaba	Kulfo‐Halaba	16	11	5	9	7	65.9 ± 5.3	53.6 ± 5.4
	Mareko	Mareko SD	17	11	6	10	7	64.2 ± 3.2	49.9 ± 8.0
South Ethiopia	Wolaita	Gindo Koisha	15	4	11	3	12	30.5 ± 1.0	18.1 ± 1.3
		Humbo	17	11	6	9	8	62.3 ± 2.1	51.5 ± 6.7
	Gofa	Bonke	18	6	12	5	13	35.5 ± 3.1	26.7 ± 8.5
		Geze‐Gofa	20	9	11	8	12	46.3 ± 7.0	38.6 ± 6.5
	Gamo	Arbaminch‐z	18	8	10	6	12	41.1 ± 6.5	31.6 ± 7.2
		Daramalo	16	10	6	8	8	63.1 ± 7.1	49.1 ± 6.7
	Ari	Jinka	14	7	7	5	9	50.2 ± 1.3	38.8 ± 2.6
	Basketo	Basketo SD	12	5	7	3	9	37.3 ± 3.2	26.5 ± 4.3
Southwest Ethiopia	Keffa	Bita	12	4	8	3	9	36.1 ± 4.7	25.0 ± 5.7
		Chena	13	4	9	3	10	33.5 ± 2.5	24.7 ± 6.4
	Bench Shako	Debub Bench	12	5	7	4	8	43.4 ± 3.3	34.3 ± 2.7
		Shako	13	5	8	3	10	34.1 ± 6.4	24.4 ± 3.4

*Note:* > 50% and < 50% cut points of highest and lowest CLS incidence intervals, whereas > 40% and < 40% are for cut points of highest and lowest CLS severity intervals.

Abbreviations: Arbaminch‐z, Arbaminch‐zuria; SD, special district; SE, standard error.

^a^ Ethiopian National Region.

^b^ Number of mungbean fields assessed for CLS.

**FIGURE 3:**
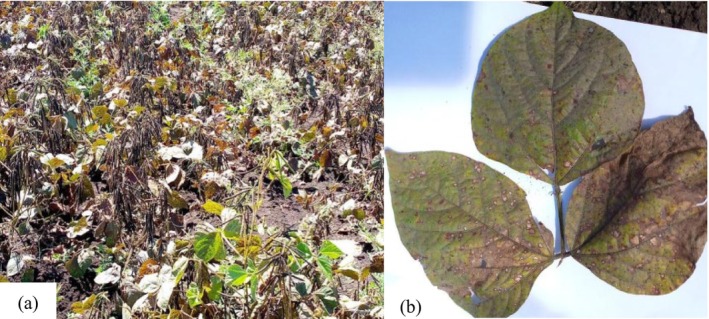
A mungbean field with (a) 100% CLS prevalence rate recorded at Sodo district, Guragae Zone of Ethiopia and (b) CLS symptom obtained from the sampled plant in 2025.

The most CLS prevalent (100%) mungbean fields were found in Sodo district. Figure 3a shows a mungbean field entirely affected by CLS in Sodo district, while Figure 3b illustrates typical CLS symptoms on leaflets collected from a sampled plant. The altitudinal elevation of the fields ranged from 731 m.a.s.l. at Gindo Koisha to 1879 m.a.s.l. at Sodo (Table S1). The majority of fields (341; 68.75%) were found situated at elevations between 1000 and 1500 m.a.s.l., followed by 84 (16.94%) fields found below 1000 m.a.s.l. The remaining 14.31% fields were situated at elevations above 1500 m.a.s.l., where CLS prevalence was highest, affecting 94.42% of fields in this range (Table 1).

Of the total 496 inspected mungbean fields, sole cropping was the predominant practice, observed in 394 fields (79.44%), followed by mixed cropping (57 fields; 11.49%) and intercropping (45 fields; 9.07%). Across the cropping systems, CLS prevalence varied, with rates of 88.53%, 96.67%, and 72.46%, respectively (Table 1). Mungbean farmers commonly practised a mixed cropping system by simultaneous cultivation of mungbean with multiple crops such as maize, sorghum, finger millet, potato and spice plants such as coriander and fenugreek in the same field simply without rows and in a less managed manner. Moreover, in a few selected districts from the Central, South and Southwest Regions of Ethiopia, mixed cropping of mungbean by farmers with already grown plants such as coffee ( *Coffea arabica* L.), khat ( *Catha edulis* L.), sugarcane ( *Saccharum officinarum* L.), and enset ( *Ensete ventricosum* L.) was observed. In addition, intercropping of mungbean was observed in a few localities across the country, including Mieso and Gemechis from West Harargae, Hageremariam Kesem and Kewot from North Shewa, Tach Gayint and Simada from South Gondar, and Tehuluader from South Wello. During the study, a distinct field observation was made between mixed cropping and intercropping of mungbean with other crops. Across all the study areas, intercropping was observed as a cropping system in which mungbean was cultivated in planned row arrangements with more deliberate management. Seed sowing practices varied across the surveyed mungbean fields. Accordingly, row sowing was practised in 40.93% of the assessed fields, while the remaining 59.07% were made through broadcast sowing with CLS prevalence rates of 84.22% and 87.03%, respectively. This indicates that sowing mungbean seeds in a row pattern is slightly preferable to broadcasting to minimize the distribution of CLS disease in mungbean production.

The soil type in each surveyed mungbean field was determined in situ based on rapid assessment of landscape position, surface cracking, and topsoil color. Fields were classified as Vertisols if they occupied flat landscapes, exhibited wide and deep surface cracks, and had dark gray to black topsoil. Conversely, fields were classified as Nitisols if they were situated on undulating or sloping terrain, showed minimal or no surface cracking, and featured dark red to reddish‐brown topsoil. Consequently, of the inspected fields, the majority (294; 59.27%) were identified as Vertisols, while the remaining 202 (40.73%) were classified as Nitisols. The mungbean plant density per quadrant (1 m × 1 m) varied from 24 to 56 plants, depending on the cropping system, either sole or mixed. The maximum mungbean plant population score (56 m^−2^) was recorded in the Geze Gofa district of the Gofa Zone and the Awash Melkassa district of the East Shewa Zone. The minimum mungbean plant density (24 mungbean plants per quadrant) was recorded in the field inspections conducted in the Shako district of the Southwest Ethiopia National Regional State, Jinka Zuria (South Ari) from the South Ethiopia National Regional State, and the Fedis district of the East Harergae Zone of Ethiopia. Across the study districts, during the varying growing seasons of the two survey years, mungbean farm fields were at three distinct phenological stages: 7.06% (35 out of 496) of fields were at the vegetative, 30.04% at the flowering, and the majority (62.90%) at the grain‐filling stages (Table 1). Correspondingly, fields at the grain‐filling stage exhibited the highest CLS prevalence (98.67%), followed by the flowering (90.36%) and vegetative stages (67.67%). These results indicate that mungbean plants are less likely to be affected by CLS before the reproductive stages. In addition, disease management actions against CLS control should begin at the flowering stage, with intimate and progressive control until maturity. In this regard, despite the prevalence of CLS, farmers in the surveyed districts did not employ any disease management practices to control or mitigate its impact on mungbean fields. This lack of intervention was informed by the farmers, who cited limited awareness of the disease, including difficulty in recognizing its symptoms. Moreover, farmers in most study districts held the misconception that CLS is not manageable, believing it originates in the soil and is aggravated by fog and humid conditions. Consequently, most mungbean producers did not take any control action against CLS disease.

**FIGURE 4:**
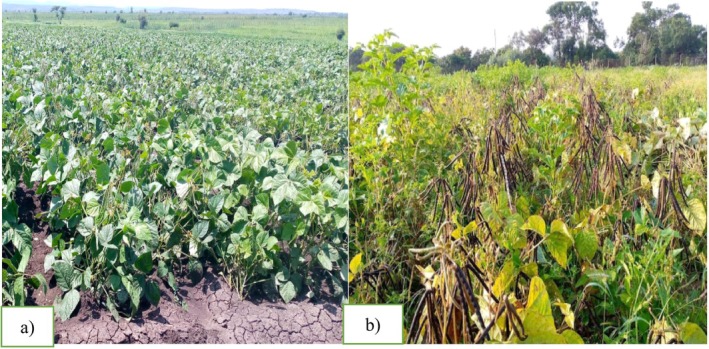
Mungbean fields: (a) weed‐free at Mieso district, (b) with high weed infestation at Awash Melkassa district of Ethiopia, during the study year of 2025.

During the inspection of mungbean fields, the level/status of weed infestation was assessed through direct observation and qualitatively rated by setting four ordinal scales as none, low, moderate, or high (Figure 4). Accordingly, only 12.90% (64/496) of the farm fields were found free of weeds; 52.82% (262/496) had low or slight weeds; 28.83% of the fields were moderately cleaned of weeds; and the remaining 5.45% were observed as highly infested with weeds. Herbaceous weeds with broad leaves were noted as the most predominant across nearly all inspected mungbean fields, while perennial grass‐like weeds with solid stems, usually known as “sedges,” were less commonly observed, and grass weeds were occasional. Based on reports from mungbean‐growing farmers across the surveyed districts, the most frequently observed weeding frequency for mungbean fields was twice per growing season.

Out of the total of 496 fields inspected, 74.80% were weeded twice, and 18.75% were weeded once. Three times or greater (≥ 3 times) weeding frequencies were observed in only 32 (6.45%) of the inspected fields, most commonly among farmers in Awash Melkassa (East Shewa Zone), Mieso (West Hararghe Zone), Tehuledere (South Wollo), Gindo Koisha (Wolaita Zone), Geze‐Gofa (Gofa Zone), and Arbaminch‐zuria. In relation to this, the occurrence of CLS disease was observed to decline as the frequency of weed cleaning increased, with prevalence rates of 97.21%, 89.72%, and 69.54% for fields weeded once, twice and ≥ 3 times, respectively. Likewise, three tillage frequencies were observed during mungbean land preparation before seed sowing: two ploughings (52.62% of fields), three ploughings (29.84% of the fields) and four ploughings (17.54%). Accordingly, mean CLS prevalence decreased progressively with increasing tillage frequency: fields plowed twice recorded the highest prevalence (94.06%), followed by those plowed three times (87.53%), while fields plowed four times exhibited the lowest prevalence (74.67%). Therefore, increasing the frequency of farmland preparation from two to four tillage events before seed sowing was associated with a 19.39% reduction in CLS prevalence. When considered incrementally, four plowing events exhibited a 12.86% reduction in CLS occurrence compared to three ploughings, and a 6.53% reduction compared to two ploughings.

During the study, substantial variations in area among the surveyed mungbean fields were noted, with the smallest (0.04 ha) recorded in Daramalo, Basketo and Chena, and the largest (approximately 3.5 ha) observed in the Bonke locality of the Gofa Zone and the Mieso district. Based on interviews with each owner of the mungbean farm fields, 349 (70.36%) fields were sown with traditional seeds obtained from the local market. The rest, 147 (29.64%), were covered by improved mungbean varieties which totally included only three improved mungbean varieties throughout all the surveyed districts, namely: Rasa (N‐26), NVL‐1 and Shewarobit. Of the identified improved mungbean cultivars, Rasa (N‐26) was found to be the most widely cultivated variety, covering 41.49% (61 out of 147) of the assessed mungbean fields, particularly in different parts of the country, including North Shewa, South Gondar, South Wello and Central Ethiopia, such as the Abeshge and Sodo districts. Additionally, 39.46% of the inspected mungbean farm fields were found to be covered by NVL‐1, mostly in East Shewa, South Ethiopia, East Harargae and Southwest Ethiopia; whereas the least, 28 out of 147 (19.05%), were covered by the Shewarobit variety. In relation to this, the lowest prevalence rate (72.52%) of CLS was observed in the mungbean farm fields with the NVL‐1 variety compared to the other two varieties, Rasa (N‐26) and Shewarobit (Table 1). The farmers also reported that for mungbean seed sowing, the local market was the dominant source, accounting for 60.28% of the farmland, followed by seeds from farmers' hands. Only 11.29% of fields were covered by mungbean seeds from agricultural offices (agri‐offices). Correspondingly, the fields sown with mungbean seeds from agri‐offices had the lowest prevalence (72.34%) of CLS (Table 1). The sowing periods of mungbean notably varied across the surveyed districts. In the Gofa, Gamo, and Wolaita zones of southern Ethiopia, sowing commonly began in mid‐February, accounting for 23.19% of the assessed fields. The majority of fields (71.17%) were sown toward the middle of July, while 5.65% were sown in early April, a common practice in the southeastern parts of Ethiopia, including the West Arsi Zone. Inorganic fertilizer application in the surveyed mungbean fields was dominated by inorganic NPS fertilizer, which was applied in 468 fields (94.35%), while the remaining fields received locally prepared organic compost. Farmers also reported that no fungicide was used in 471 of the assessed mungbean fields, accounting for 94.96% of the total surveyed farm fields, and that there was no notable variation in CLS prevalence among them. Over half (52.22%) of the mungbean fields assessed were affected by waterlogging. The prevalence of CLS was notably higher in waterlogged fields (96.33%) than in non‐waterlogged fields (74.07%), as shown in Table 1. This indicates that waterlogging may create conditions conducive to CLS development in mungbean fields, depending on other interacting factors such as plant density and humidity.

### 3.2 Incidence and Severity of CLS in Major Mungbean‐Growing Districts of Ethiopia

Even though varying levels of mean CLS incidence and severity were recorded across the 33 surveyed districts, in nearly all districts, both overall means of CLS PDI and PSI were also higher in 2025 (52.3 ± 1.3; 42.1 ± 0.5) than in 2024 (47.8 ± 2.3; 38.2 ± 3.3), in that order (Table 1). Furthermore, a clear altitudinal trend was observed where fields at high altitudes (> 1500 m.a.s.l.) recorded the highest disease incidence (61.2%) and severity (50.4%), significantly exceeding those at mid (1000–1500 m.a.s.l.) and low altitudes (< 1000 m.a.s.l.). Among the cropping systems assessed, CLS incidence and PSI were highest in mixed cropping systems (61.7% and 52.6%, respectively), followed by sole cropping (52.1% and 40.0%) and intercropping (31.5% and 19.5%). Notably, intercropping reduced CLS incidence and severity by 30.2% and 33.1%, respectively, relative to mixed cropping (Table 1). Fields established by row sowing exhibited significantly lower CLS occurrence than those established by broadcasting. Moreover, the incidence and severity of CLS in the inspected fields across the 33 localities were assessed based on the variable category of soil type. However, there were only slight numerical differences between the two soil types (Vertisol and Nitisol) in their levels of incidence or PSI of CLS, with the nearly similar mean CLS incidence of 48.4 ± 6.4 versus 51.7 ± 4.1 and PSI of 39.7 ± 4.0 versus 41.0 ± 2.1, respectively. Higher disease incidence (61.4%) and PSI (52.4%) were recorded from the farm field with densely planted (> 40 plants m^−2^) fields than those with thinly planted fields. Farms at the grain‐filling and flowering growth stage showed much higher disease incidence and severity than those at the vegetative stages (Table 1). The status of the inspected mungbean fields was also assessed based on weed infestation levels. Accordingly, clean (weed‐free) mungbean farms were observed, with slight CLS (32.7% incidence and PSI of 24.0%). In contrast, fields with high weed infestation recorded substantially higher values (64.3% incidence and 56.1% PSI). Across all districts and both study years, CLS intensity was greater in farms that received less frequent weeding (once) compared to those that were well‐weeded (≥ 3 times). These findings indicate that maintaining weed‐free conditions and implementing regular weeding practices can reduce CLS pressure by more than half in mungbean fields.

Tillage frequency prior to sowing was evaluated for its effect on incidence and severity of CLS in mungbean farms. Fields plowed twice recorded the highest disease incidence (62.7 ± 5.2) and severity (48.0 ± 3.0), whereas fields tilled four times exhibited the lowest incidence (38.7 ± 4.5) and severity (31.5 ± 5.5). Larger mungbean fields (> 0.25 ha) exhibited slightly higher mean CLS incidence (52.5%) and PSI (41.1%) relative to smaller fields (incidence = 49.4% and PSI = 38.2%) (Table 1).

Mungbean fields planted with traditional cultivars showed considerably higher mean CLS incidence (60.3%) and percentage severity index (PSI) (48.2%) compared to those sown with improved varieties (39.5% and 28.1%, respectively). However, the local varieties exhibited variable responses to CLS across the inspected fields. During the study, the names of the known improved mungbean varieties commonly used by farmers across the inspected farms were also recorded to assess the intensity of CLS occurrence in relation to these varieties. As a result, across all the mungbean farms surveyed in 33 districts of Ethiopia, only three improved varieties were found in cultivation: N‐26, NVL‐1 and Shewarobit. CLS incidence and severity varied considerably among these varieties across the localities. The lowest CLS incidence (37.0 ± 1.5) and PSI (26.7 ± 3.5) were noted on variety NVL‐1, followed by N‐26 (incidence = 44.1 ± 0.6, PSI = 35.2 ± 0.4), while the highest values were observed on Shewarobit (incidence = 68.3 ± 4.2, PSI = 59.0 ± 6.1) (Table 1). This is evidence that using a relatively resistant variety might help minimize the occurrence of CLS in mungbean fields. Fields planted with seeds purchased from local markets recorded the highest CLS incidence (69.6%) and PSI (62.7%), followed by those established with seeds obtained from neighboring farmers. In contrast, fields sown with seeds supplied by agricultural offices exhibited substantially lower incidence (33.2%) and PSI (24.7%). Disease incidence and severity were also strongly influenced by sowing time, with mid‐July plantings showing the highest CLS incidence (60.7%) and PSI (52.2%), whereas early‐planted fields consistently exhibited lower disease levels across all surveyed localities. Similarly, fields receiving recommended inorganic fertilizer applications had markedly lower CLS incidence (35.4%) and PSI (25.3%) than nutrient‐deficient fields, which recorded 63.1% incidence and 54.2% PSI (Table 1), indicating the potential role of balanced nutrition in enhancing host tolerance to CLS.

Among the inspected mungbean‐growing districts, the highest CLS incidence (72.4 ± 3.4) and PSI (67.0 ± 2.2) were observed in the Sodo district, followed by Adami Tullu, Awash Melakassa, Kulfo‐Halaba and Lebo Kemkem. On the other hand, the lowest mean incidence and severity (PSI) values for CLS were recorded in Mieso (28.6 ± 1.1 and 17.3 ± 1.4) and Gindo Koisha (30.5% and 18.1%), respectively (Table 2).

Although fungicide‐treated fields showed lower disease incidence and severity than untreated fields, fungicide use was uncommon, being reported in only 5% (25 of 496) of surveyed fields. Moreover, except in six districts (Awash Melkassa, Adami Tullu, Arsi Negele, Mieso, Geze Gofa, and Gindo Koisha), growers did not apply fungicides for CLS management, largely because specific fungicides were unavailable. Among the few (5%) fungicide user farmers, mancozeb was the only fungicide commonly reported for CLS management practices. Moreover, most farmers lacked knowledge of the appropriate crop growth stage and timing for fungicide application, alternative effective fungicides, and integrated disease management practices. This can indicate that CLS management in mungbean production using chemical fungicide is still at an early stage of adoption. Instead of using chemical fungicide, mungbean growers in different parts of the study districts indicated that they have been using different ways of CLS management practices such as the use of relatively less susceptible varieties such as NVL‐1 and N‐26, use of seed from agricultural offices, crop rotation, field sanitation and residue removal through frequent tillage, and timely sowing.

### 3.3 Association of CLS in Mungbean With Agronomic Factors

Table 3 summarizes the results of the logistic regression analysis using the likelihood ratio test, indicating the association between the agronomic factors (independent variables) and the incidence and severity of CLS in mungbean‐growing localities of Ethiopia during the 2024 and 2025 cropping seasons. The logistic regression analysis revealed that 15 out of 19 agronomic factors, including “district” as an independent variable, exhibited a statistically highly significant association (*p* < 0.0001) with both CLS incidence and severity when added at first (Analysis I) and last (Analysis III) into the model (Table 3). This indicates that the 15 agronomic factors studied here can be considered key predictors of CLS occurrence across the districts in Ethiopia. Considering the district as one of the independent variables, the results of the logistic regression analysis indicated that there was statistically significant variation in terms of both CLS incidence and PSI among the surveyed mungbean‐growing districts (Table 3). For instance, in Analysis I (Type I) based on the first entered variables (*V*_1_) in the model, the assessed CLS incidence showed highly significant (*χ* ^2^ = 694.3, *p* < 0.0001) variation among the surveyed districts. Among the three variable categories of the cropping system, there was strong significant variation to predict both CLS incidence (*χ* ^2^ = 798.4, *p* < 0.0001) with highest deviance reduction (*D*↓) 6219.04 and CLS severity (*χ* ^2^ = 512.7, *p* < 0.0001) with highest *D*↓ of 5879.5 next to that of district and year, both in Analysis I (*V*_1_). CLS incidence and severity differed significantly among tillage practices (*χ* ^2^ = 404.5, *p* < 0.0001 and *χ* ^2^ = 223.2, *p* < 0.0001, respectively), Table 3. These findings suggest that increased tillage frequency can substantially reduce CLS intensity in mungbean production. Similarly, in Analysis III (*V*_2_), the cropping system continued to reveal statistically significant variation across its variable categories, predicting both CLS incidence and severity. The mean CLS incidence across the two assessed sowing patterns (row and broadcasting) showed highly significant variation (*χ* ^2^ = 249.3, *p* < 0.0001) in Analysis I when *V*_1_ (first entered) was included in the model. However, its significance level for predicting CLS incidence in mungbean decreased to *p* < 0.0741 when entered last in Analysis III (*V*_2_) with the deviance value reduced from *D*↓ = 1165.7 to *D*↓ = 74.2. The severity (PSI) of CLS for these sowing patterns varied significantly, *χ* ^2^ = 124.5, *p* < 0.0173 (Table 3). The agronomic factors that showed significant associations with higher deviance reduction (*D*↓) and lower Chi‐square (*χ* ^2^) values were considered predictor variables and added to the logistic regression model. This further indicated their associations and importance in influencing the development of CLS in the mungbean production system.

**TABLE 3:** Logistic regression model of likelihood ratio test for incidence and severity of CLS in mungbean based on the independent variables assessed during the 2024 and 2025 cropping seasons in Ethiopian.

Variable	df	CLS incidence (PDI)					CLS severity (PSI)				
		*χ* ^2^	Analysis I (*V*_1_)		Analysis III (*V*_2_)		*χ* ^2^	Analysis I (*V*_1_)		Analysis III (*V*_2_)	
			*D*↓	*p* > *χ* ^2^	*D*↓	*p* > *χ* ^2^		*D*↓	*p* > *χ* ^2^	*D*↓	*p* > *χ* ^2^
Districts	32	694.3	7445.2	< 0.0001	657.7	< 0.0001	436.0	7148.5	< 0.0001	263.7	< 0.0001
Year	1	521.1	6769.4	< 0.0001	322.6	< 0.0001	354.8	6399.6	< 0.0001	144.6	< 0.0001
Altitude	2	712.6	5503.57	< 0.0001	346.5	< 0.0001	478.8	5311.3	< 0.0001	175.6	< 0.0001
Cropping system	3	798.4	6219.04	< 0.0001	216.5	< 0.0001	512.7	5879.5	< 0.0001	191.6	< 0.0001
Sowing pattern	1	249.3	1165.7	< 0.0001	74.2	0.0741	124.5	90.8	0.0173	14.7	0.6575
Soil type	1	360.5	351.4	0.0031	96.7	0.0822	187.4	117.6	0.0074	42.0	0.8015
Plant density	1	512	5367.87	< 0.0001	213	< 0.0001	343.4	5174.8	< 0.0001	122.3	< 0.0001
Plant growth stage	2	294.6	4293.5	< 0.0001	207.2	< 0.0001	209.2	4106.4	< 0.0001	145.4	< 0.0001
WIL	3	403.3	4976.4	< 0.0001	218.7	< 0.0001	270.3	4798.8	< 0.0001	150.0	< 0.0001
Weeding frequency	2	367.7	5194.43	< 0.0001	226.4	< 0.0001	243.4	5009	< 0.0001	127.3	< 0.0001
Tillage	2	404.5	4869.7	< 0.0001	269.6	< 0.0001	223.2	4469.7	< 0.0001	210.4	< 0.0001
Field size	1	197.3	689.4	0.0171	59.4	0.3601	103.1	78.2	0.0435	7.6	0.8771
Mungbean cultivar	1	377.1	4112.49	0.0042	220.5	< 0.0001	214	3988.3	< 0.0001	124.3	< 0.0001
CI varieties	2	311.7	4537.16	< 0.0001	186.3	< 0.0001	208.2	4397.2	< 0.0001	139.5	< 0.0001
Seed Source	2	244.4	3623.23	< 0.0001	165.5	< 0.0001	171.1	3467.2	< 0.0001	112.3	< 0.0001
Sowing time	2	186.2	4489.05	< 0.0001	149	< 0.0001	125.3	4279.6	< 0.0001	120.1	< 0.0001
Fungicide application	1	136.7	1012.6	0.0169	67.7	0.2714	100.7	69.6	0.0041	32.8	0.0483
Inorganic fertilizer	1	421.3	1781.7	< 0.0001	129.5	< 0.0001	274.9	1659.7	< 0.0001	87.1	< 0.0001
Water logging	1	203.5	2043.05	< 0.0001	141.3	< 0.0001	132.5	2001.5	< 0.0001	106.1	< 0.0001

Abbreviations: CI varieties, cultivated improved varieties; *D*↓, deviance reduction; df, degrees of freedom; *V*_1_, variable added first during type I analysis; *V*_2_, variable added last during type III analysis; *p*, probability of an *χ* ^2^ value greater than the value of *D*↓; WIL, weed infestation level; *χ* ^2^, Chi‐squared.

On the other hand, four of the agronomic factors, namely sowing pattern, soil type, farm size and fungicide application, showed no significant association with either incidence or severity of CLS, and hence were not entered into the model. The outcomes of logistic regression analysis for each predictor variable and variable category with respect to likelihood ratio tests including the intercept (null deviance), residual deviance, deviance reduction (*D*↓), model estimate (ME), standard error (SE) and odds ratio (OR) were estimated for both incidence (Table 4) and severity (Table S2) of CLS. Considering Mieso district as a reference district, the probability of a high CLS incidence (in the cut point of ≥ 50%) in Awash Melkassa, Adami Tullu, Seraro, Gemechis, Lebo Kemkem, Kallu, Sodo, Kulfo‐Halaba, Mareko SD, Humbo, and Daramalo was 2.53, 2.68, 2.14, 2.28, 2.38, 2.44, 3.04, 2.61, 2.40, 2.46 and 2.32 times higher, respectively, than the CLS incidence in Mieso district (Table 4). Besides, mungbean fields cultivated during the 2024 cropping season and located at altitudinal ranges of ≤ 1000 m.a.s.l. showed 7% and 21% lower CLS incidence, respectively, than the farm fields in 2025 at an altitude of 1000–1500 m.a.s.l. (the reference altitude). However, the mungbean fields cultivated at altitudinal ranges above 1500 m.a.s.l. had a 1.21 times higher CLS incidence than those cultivated at the reference altitude (Table 4). Fields cultivated with the mixed cropping system exhibited a 1.17 times higher CLS incidence than the mungbean fields cultivated with sole cropping, whereas the intercropping variable category revealed 41% lower probability of risk of CLS disease occurrence compared with that of sole cropping. Plant density of ≤ 40 plants per m^2^, and the categorical variables of plant growth stage, such as vegetative and flowering growth stages, showed 34%, 52%, and 20% lower probability of risk of CLS incidence, respectively, than the plant density of ≥ 40 plants per m^−2^ and grain‐filling growth stage. Moderate and high levels of weed infestation, less frequent weeding practice, less frequent tillage, the unavailability of an improved mungbean cultivar such as NVL‐1, lack of inorganic fertilizer and presence of waterlogging, showed a higher association with a high likelihood of CLS incidence than their corresponding variable category in each of their entered predictor variables (Table 4).

**TABLE 4:** Outcomes of logistic regression analysis of the likelihood ratio test for predictor variables and variable categories with respect to CLS incidence in mungbean‐growing areas of Ethiopia, during the 2024 and 2025 cropping seasons.

Entered variable	Likelihood ratio test for CLS incidence							
	Residual *D*	df	*D*↓	*p* > *χ* ^2^	V. category	ME	SE	ORs
Intercept	9754.3	—	—	—	Intercept	−0.417	0.1779	0.67
District	2309.1	32.0	7445.2	< 0.0001	Awash Melkassa	0.614	0.0198	2.53
					Adami Tullu	0.654	0.0136	2.68
					Arsi Negelle	0.410	0.0614	1.90
					Seraro	0.425	0.0339	2.14
					Mieso	0*	—	1.00
					Gemechis	0.527	0.0328	2.28
					Fedis	−0.406	0.0846	1.27
					Babile	−0.175	0.6419	1.90
					Merhabete	0.517	0.0685	2.17
					Minjarna Shenkora	0.405	0.0687	1.90
					Hageremariam K	−0.275	0.0714	1.56
					Kewot	0.406	0.0577	1.90
					Lebo Kemkem	0.576	0.0352	2.38
					Tach Gayint	0.528	0.0263	2.17
					Simada	0.411	0.0595	2.03
					Kallu	0.529	0.0347	2.44
					Tehuluader	−0.625	0.0813	1.17
					Abeshge	0.409	0.0627	2.11
					Sodo	0.747	0.0125	3.04
					Kulfo‐Halaba	0.581	0.0339	2.61
					Mareko SD	0.578	0.0475	2.46
					Gindo Koisha	−0.715	0.0977	1.03
					Humbo	0.531	0.0489	2.40
					Bonke	−0.413	0.0873	1.27
					Geze‐Gofa	−0.618	0.0143	1.71
					Arbaminch‐zuria	−0.285	0.0751	1.69
					Daramalo	0.537	0.0468	2.32
					Jinka	0.402	0.0696	1.90
					Basketo SD	−0.357	0.0813	1.58
					Bita	−0.365	0.0867	1.27
					Chena	−0.423	0.0895	1.17
					Debub Bench	−0.321	0.0732	1.58
					Shako	−0.397	0.0882	1.46
Year	1169.2	1	6769.4		2024	−0.079	0.0701	0.93
					2025	0*	—	1.00
Altitude	1265.8	2	5503.6	< 0.0001	≤ 1000	−0.075	0.0856	0.79
					1000–1500	0*	—	1.00
					≥ 1500	0.840	0.0073	1.21
Cropping system	715.5	2	6219.1	< 0.0001	Sole cropping	0*	—	1.00
					Mixed cropping	0.852	0.0057	1.17
					Intercropping	−0.055	0.0849	0.59
Plant density	912.7	1	5367.9	< 0.0001	≤ 40 m^−2^	0.740	0.0082	0.66
					> 40 m^−2^	0*	—	1.00
Plant growth stage	531.6	2	4293.5	< 0.0001	Vegetative	−0.031	0.0913	0.48
					Flowering	0.451	0.0095	0.80
					Grain‐filling	0*	—	1.00
Weed infestation level/status	465.2	3	4976.4	< 0.0001	None	−0.042	0.0787	0.71
					Low	0*	—	1.00
					Moderate	0.765	0.0086	1.26
					High	0.81	0.0059	1.36
Weeding frequency	398.5	2	5194.4	< 0.0001	Once	0.876	0.0025	1.93
					Twice	0*	—	1.00
					≥ 3 times	−0.047	0.0675	0.96
Tillge	498.7	2	4869.7	< 0.0001	2 times	0.799	0.0079	1.35
					3 times	0*	—	1.00
					4 times	−0.085	0.0961	0.93
Mungbean cultivar	304.3	1	4112.5	< 0.0001	Improved	0*	—	1.00
					Traditional	0.762	0.0056	0.56
CI varieties	411.4	2	4537.2	< 0.0001	Rasa (N‐26)	0*	—	1.00
					NVL‐1	−0.0511	0.0265	0.82
					Shewarobit	0.855	0.0045	1.54
Seed Source	231.0	2	3623.2	< 0.0001	Agri‐offices	−0.032	0.075	0.49
					Local market	0*	—	1.00
					Farmers	0.816	0.0036	0.68
Sowing time	376.7	2	4489.1	< 0.0001	Mid‐February	−0.062	0.0224	0.72
					Early April	−0.067	0.0143	0.46
					Mid‐July	0*	—	1.00
Inorganic fertilizer	146.4	1	1781.7	< 0.0001	Applied	−0.022	0.0567	1.83
					Not applied	0*	—	1.00
Water logging	187.36	1	2043.1	< 0.0001	Absent	0*	—	1.00
					Present	0.881	0.0057	2.67

Abbreviations: *D*↓, deviance reduction; df, degrees of freedom; ME, coefficient of model estimate (stable model estimates); *p*, probability of *χ* ^2^ value greater than the value of *D*↓; Residual *D*., residual deviance; SE, standard error; V. category, variable category; *χ* ^2^, Chi‐squared; *, reference group (the category with an odds ratio, OR, of 1.00).

CLS severity at the 40% (≥ 40%) cut point had the highest probability of occurrence and showed a highly significant association with several districts. The remarkable odds ratio of CLS severity were observed in Awash Melkassa, Adami Tullu, Seraro, Gemechis, Lebo Kemkem, Tach Gayint, Simada, Kallu, Sodo, Kulfo‐Halaba, Mareko SD, Humbo, and Daramalo at 2.90, 3.07, 2.37, 2.53, 2.97, 3.05, 3.17, 2.67, 2.79, 2.51, and 2.38 times greater, respectively, than the reference district, Mieso (Table S2). During the 2025 cropping season, the likelihood of CLS severity manifestation was 12% greater than that of 2024 in the ≥ 40% interval, while it was 27% lower for mungbean farms situated at altitudes ≤ 1000 m.a.s.l. On the other hand, a 30% greater likelihood was observed for fields cultivated at an altitude of 1500 m.a.s.l., compared to fields at an altitude of 1000–1500 m.a.s.l. (Table S2). The predictor variable, the cropping system of mungbean farms, revealed a strong association with CLS severity (≥ 40%). Compared to the farms managed with sole cropping, mixed cropping was associated with a 31% increase in CLS severity, whereas intercropping was associated with a 50% reduction in CLS severity risk. At the > 40% severity cut point, CLS severity was associated with lower risks across several mungbean management and field condition categories.

Compared to their respective reference categories, lower risks of CLS were observed for plant density (< 40 m^−2^), vegetative stage, flowering stage, weed‐free (none) fields, frequent weeding (> 3 times), increased tillage and use of improved cultivars, with risk reductions of 47%, 66%, 21%, 30%, 16%, 12%, and 55%, respectively (Table S2). Mungbean seeds obtained from agricultural offices and farmers' stocks were associated with 47% and 39% lower severity, respectively, compared with mid‐July. The absence of inorganic fertilizer and the presence of waterlogging in the mungbean fields were associated with the highest CLS severity, with odds ratio of 2.06 and 2.75, respectively, compared with farms with fertilizer application and those free from waterlogging (Table S2).

## 4 Discussion

The present study provides the first comprehensive assessment of CLS distribution and occurrence across major mungbean‐growing districts in Ethiopia. CLS was found to be widely distributed across the surveyed mungbean‐growing districts of Ethiopia, with varying prevalence, incidence and severity. The results of the present study confirm that CLS is a widespread and economically important disease, with a prevalence of more than 85% in most surveyed districts and a mean incidence and severity of 50% and 40%, respectively (Figure 2, Tables 1 and 2). These results align with global reports indicating that CLS, caused by *C. canescens* Ellis & Martin, is the most destructive fungal disease affecting mungbean production worldwide (Irfan et al. 2025; Inthaisong et al. 2025; Pattnaik et al. 2024; Gata et al. 2024; Mota et al. 2021; Dikr 2023; Nair et al. 2019). The observed variation in the occurrence of CLS in Ethiopia could be attributable to changing agro‐ecological factors, including altitudinal ranges, climatic components, soil types and different crop management systems in the studied areas. The widespread distribution of CLS across diverse agro‐ecologies in Ethiopia can indicate the pathogen's adaptability and the vulnerability of current production systems (Dikr 2023; Gata et al. 2024). Over half (54.55%, or 18 out of 33) of the assessed districts exhibited CLS severity above the grand mean (> 40%). Most mungbean‐growing areas in Ethiopia face conditions favorable to CLS, underscoring the critical need to prioritize and implement effective CLS management strategies in these regions. CLS remains a significant bottleneck in mungbean production worldwide, particularly in mungbean‐growing areas of Ethiopia (Dikr 2023; Umata 2018).

Increasing trends in the prevalence, incidence and severity of CLS were observed in the 2025 cropping season compared to 2024 (Figure 2, Table 1). Such variations among the seasons might be due to favorable agro‐ecological factors in 2025, characterized by high relative humidity, frequent rainfall and ideal temperatures, which were more conducive to CLS pathogen sporulation, dispersal and infection compared to the relatively drier and less favorable conditions observed in 2024. This finding is consistent with those of Chand et al. (2025); Yadav et al. (2024), who reported high CLS incidence (often > 60%) with clear spatial variation across locations and years of mungbean production, reflecting strong environmental and management practices. Even though almost all of the potential mungbean‐growing districts included in the present study were from the lowland and mid‐land areas (780–1870 m.a.s.l.) of the country (Table S1), higher CLS incidence and severity were observed in farms located above 1500 m.a.s.l. than in farms below 1500 m.a.s.l. This implies that the risk of CLS pressure in mungbean increases with increasing altitude within this narrow range. This finding can be explained by the fact that higher altitudes typically experience lower mean temperatures and higher relative humidity, which prolong leaf wetness duration, thereby favoring CLS progression (Irfan et al. 2025; Khaire et al. 2024; Das et al. 2019). This finding is in agreement with (Dikr 2023) who described mungbean as a warm‐season, lowland legume adapted to elevations of 1000–1650 m.a.s.l., with 600–750 mm annual rainfall and temperatures around 27°C–30°C in Ethiopia. Optimal development for CLS pathogen conidia occurs at 25°C–30°C and high relative humidity, creating ideal conditions for CLS outbreaks in mungbean (Kumar et al. 2020; Das et al. 2019).

The present study revealed that mungbean appears less vulnerable to CLS before the reproductive stage, in which both incidence and severity of CLS increase consistently with the advancement of crop growth, from the vegetative stage through flowering and pod setting to the grain‐filling stage (Table 1). Accordingly, effective disease management should begin at flowering and be sustained progressively throughout the remainder of the cropping cycle. This could be attributed to the combination of mungbean plant physiological changes, biochemical defense dynamics and patterns of disease development in field studies. This finding is consistent with findings of the studies by Sahoo et al. (2022), Mahapatra et al. (2022), and Choudhary and Chand (2022), who confirmed that mungbean plants are vulnerable during reproductive stages, when photosynthetic efficiency is already under demand as the plant diverts significant metabolic resources toward flower and pod development. CLS incidence and severity were lower in intercropping than in both mixed and sole‐cropping systems (Table 1). This can be due to intercropping with a controlled, systematic row arrangement, which creates gaps or physical barriers that prevent airborne CLS spores from reaching mungbean foliage and improve air circulation, reducing leaf wetness duration. A mixed‐cropping system can reduce irregular mungbean leaf canopy closure, air flow and high humidity, resulting in high CLS risk. The duration of leaf wetness in a mungbean field is a key factor favoring the development of the CLS pathogen (Bughio 2017). A previous study by Singh and Singh (2014), confirmed that intercropping mungbean with cereals such as sesamum, sorghum or millet showed synergistic effects, improving plant health and yield by significantly reducing the incidence of CLS compared to sole cropping. Additionally, intercropping mungbean with non‐host crops increases the distance between mungbean plants across fields, thereby reducing the probability of spore transmission. Dense, uniform host plant populations in sole cropping facilitate rapid CLS spread and epidemic severity (Kumar et al. 2020).

Across the assessed mungbean‐growing districts, fields with fewer mungbean plants (< 40 plants per m^2^) were also associated with lower CLS incidence and severity (Table 1). Compared to mungbean fields with lower plant density, fields with densely cultivated mungbean plants had denser canopies because the physical distance between host plants was minimal. This could create conditions, such as a humid microclimate with reduced air circulation and prolonged leaf wetness that favor the progression and further spread of CLS. This is consistent with (Douma and Noordhoek 2025) who synthesized data from 44 studies and concluded that higher host planting density increases disease occurrence, probably due to higher pathogen spread and minimized plant defense. Across the assessed mungbean‐growing districts in this study, less frequently and poorly weeded mungbean fields revealed significant CLS incidence and severity (Table 1). This could be because weed infestation increases overall canopy density and reduces air circulation under the crop canopy, increasing leaf wetness and trapping humidity, which can lead to conidial germination of the CLS pathogen. Conversely, fields with low weed infestation have more open canopies, better air flow and shorter periods of leaf wetness, which are less favorable for CLS development. In addition, infected weeds act as a green bridge, producing CLS spores that serve as the primary inoculum for the next mungbean crop. As the pathogen causing CLS is not host‐specific (Shahzady et al. 2017; Chauhan et al. 2022), spores produced on weeds can be wind‐dispersed or splashed by rain onto mungbean plants, initiating new infections throughout the growing season. Regular weeding removes potential alternative hosts from the field, breaking the disease cycle by eliminating the green bridge that allows the pathogen to persist and multiply between mungbean cropping cycles or even within a single season. The finding is consistent with studies by Meena et al. (2024), on the integrated management of foliar diseases of mungbean under natural field conditions. It was also observed that mungbean plants cultivated on farm fields with reduced tillage practices were associated with a higher incidence and severity of CLS. This may be attributed to the survival of the CLS pathogen in infected crop residues remaining on the soil surface, whereas frequent tillage accelerates residue decomposition and reduces fungal inoculum availability. This is consistent with the findings of Meena et al. (2024). These findings are also consistent with those reported by Mengistu et al. (2015) who showed that reduced or no‐tillage promotes the persistence of infected crop residues on the soil surface, increasing pathogen survival and disease development. Similarly, Orrù et al. (2021) demonstrated that tillage practices influence soil fungal communities, including the abundance of plant‐pathogenic fungi.

Three mungbean seed sources were identified across the study districts as certified improved seed obtained from agricultural offices such as NVL‐1 and Rasa, farmer‐saved seed maintained through informal community exchange, and uncertified seed purchased from local markets with variable varietal identity and purity. These differences in varietal characteristics and seed quality may influence field establishment, plant health, and ultimately the occurrence and severity of CLS. This finding is consistent with that reported by (Kassa et al. 2022). Mungbean fields planted with the improved mungbean varieties brought from agricultural offices were more strongly associated with a lower incidence and severity of CLS than those planted with traditional varieties or purchased from local markets, across almost all the surveyed districts of Ethiopia. This might be due to differences in mungbean cultivars' responses to CLS infection, resulting from genetic variations that direct their biochemical defense systems (Chankaew et al. 2021). In line with the results of the present study, Sahoo et al. (2022) confirmed that improved mungbean varieties possess genetically determined resistance traits, including higher phenolic content and enhanced activity of defense‐related enzymes, negatively correlated with CLS severity. The same authors indicated that, in contrast, traditional seeds lack systematic selection for resistance and may harbor heterogeneous susceptibility levels to CLS. Additionally, as CLS is a seed‐borne disease, mungbean seeds obtained from agricultural offices are normally subjected to health testing and certification, ensuring they are free of seed‐borne inocula. CLS incidence and severity varied significantly across the identified mungbean varieties, with the lowest disease level recorded in NVL‐1 followed by that of N‐26, and the highest in Shewarobit (Tables 1 and 3). Across most of the study areas, it was also noted that N‐26 (Rasa) had superior yield, large seed size, and farmer preference; NVL‐1 had longer pods with medium deep green seeds having longer thin dark green leaves and relatively not affected by disease. In contrast, Shewarobit, despite its early maturity and drought tolerance, easily indicate some foliar infections, had smaller seeds and lower market demand. These varieties differ in their genetic background, maturity period, yield potential, and response to biotic and abiotic stresses, including susceptibility to CLS. These findings are partially consistent with previous reports describing variation in agronomic performance and disease response among improved mungbean varieties in Ethiopia (Kassa et al. 2018; Dinsa et al. 2022; Delie et al. 2025).

Both the lower incidence and severity of CLS were observed across almost all the inspected mungbean fields treated with balanced inorganic fertilizer. The lower CLS incidence and severity observed in fertilized fields may be associated with improved plant nutrition through fertilizer application, which can enhance plant vigor and strengthen structural and biochemical defense mechanisms against CLS pathogen infection. However, though the influence of inorganic fertilizers on disease development depends on the type, rate, and timing of nutrient application, as well as the host–pathogen interaction. This finding is in agreement with that of Singh and Singh (2014), who reported the application of inorganic fertilizer, combined with other methods, plays a significant role in managing of CLS in mungbean by enhancing plant vigor, increasing resistance and yield. These findings are also consistent with those of (Tripathi et al. 2022) who reported that balanced mineral nutrition, including adequate supplies of N, P, K, Ca, Si, Mn, and Zn, enhances plant defense and may reduce disease severity. Similarly, (Weinmann et al. 2023) explained that adequate mineral nutrition strengthens cell walls, promotes the synthesis of defense‐related compounds, and activates plant immune responses, thereby enhancing resistance to pathogen infection.

Moreover, mungbean farms free from waterlogging exhibited a highly significant relationship with reduced incidence and severity of CLS (Table 1). This could be due to the absence of waterlogging, which reduces CLS pressure by limiting the duration of leaf wetness and splash dispersal of spores. In the present study, fungicide application on mungbean farms did not show a significant effect on the intensity (PDI and PSI) of CLS disease (Table 3). This absence of a significant association between fungicide application and CLS intensity (PDI and PSI) may be due to the limited adoption of chemical fungicide across most of the study areas, where chemical disease management remains at an early stage. This finding contradicts previous reports by Singh and Singh (2014) and Meena et al. (2024), which demonstrated that integrated chemical management significantly reduced CLS incidence in mungbean. The difference between the findings of the present study and that of the previous ones could be due to differences in study design and management conditions. The previous studies were conducted under controlled experimental conditions using integrated disease management packages, whereas the present study was a farmer field–based survey in which fungicide application was uncommon and, where practiced, was generally not integrated with other recommended management practices. Moreover, differences in agro‐ecological conditions, cropping systems, and pathogen populations between study regions and countries may have further contributed to the contrasting outcomes. Consequently, the effectiveness of fungicide use under small holder farmers' production systems may not have been sufficient to produce a measurable reduction in CLS intensity.

## 5 Conclusion

The present study demonstrates that CLS is widely distributed across major mungbean‐growing areas of Ethiopia, confirming its economic importance and threat to mungbean productivity and food security. The study further shows that CLS incidence and severity varied with the assessed agronomic factors, such as districts, altitude, cropping system, crop growth stage, tillage frequency, weed infestation status, regularity of weeding, seed sources, sowing time, availability of adequate resistant cultivars and application of inorganic fertilizers. These were significantly associated with CLS incidence and severity across seasons. They were identified as key predictors of CLS occurrence across the study districts. Integrated CLS management is therefore essential, with emphasis on high‐risk districts and practices such as land preparation, intercropping, optimized sowing time, resistant cultivars, balanced fertilization and effective management of waterlogging, weed, and plant density management. The non‐significant factors in this study, such as farmland size, soil type, and sowing pattern, may require further investigation to understand their limited role under the diverse agro‐ecological conditions of mungbean‐growing systems in Ethiopia. This study also confirmed the need to strengthen plant health extension services to improve farmers' awareness and adoption of CLS management practices in the mungbean production system, including the use of appropriate alternative fungicides when necessary. In the present study, mungbean fields with heavy mixed infections, ambiguous symptoms, where other pathogens contributed > 20% of foliar damage were not considered. Therefore, future controlled co‐inoculation studies with molecular diagnostics are needed to evaluate synergistic effects on disease and yield, complementing our spatial survey and aiding management strategies.

## Funding

A modest fund for field data collection was provided by Madda Walabu University, Ethiopia, with the reference number of AT‐27/123/566.

## Conflicts of Interest

The authors declare no conflicts of interest.

## Supporting information


**Table S1:** Altitudinal ranges, number of mungbean fields inspected per district, absolute locations of study districts and weather conditions of CLS surveyed districts in Ethiopia during the 2024 and 2025 cropping seasons.

**Table S2:** Outcomes of logistic regression analysis of the likelihood ratio test for predictor variables and variable categories with respect to CLS severity in mungbean‐growing areas of Ethiopia, during the 2024 and 2025 cropping seasons.

## Data Availability

The data that support the findings of this study are available on https://doi.org/10.25399/UnisaData.32946833.
