# Supplementary material for: Distribution and Occurrence of Cercospora Leaf Spot of Mungbean (Vigna radiata) and Associated Agronomic Factors in Ethiopia
Source: Plant Environ Interact. 2026 Jul 24;7(4):e70194. doi: 10.1002/pei3.70194 (PMC13400991; doi:10.1002/pei3.70194)
Supplement: Supplementary file 1 — Table S1: Altitudinal ranges, number of mungbean fields inspected per district, absolute locations of study districts and weather conditions of CLS surveyed districts in Ethiopia during the 2024 and 2025 cropping seasons. [file PEI3-7-e70194-s001.docx]

Supplementary Table 1 (Table S1) | Altitudinal ranges and weather conditions of CLS surveyed areas in Ethiopia during the 2024 and 2025 cropping seasons

| Region^*^ | Zone | Districts/ Localities | Number of fields | Location | | Altitude (m.a.s.l.) | mRF (mm) | | Temperature (°C) | | | | RH (%) | |
| --- | --- | --- | --- | --- | --- | --- | --- | --- | --- | --- | --- | --- | --- | --- |
|  |  |  |  | Latitude (N) | Longitude (E) |  | 2024 | 2025 | 2024 | | 2025 | | 2024 | 2025 |
|  |  |  |  |  |  |  |  |  | Min | Max | Min | Max |  |  |
| Oromia | East Shewa | Awash Melkassa | 18 | 08° 26' 00" | 39° 13' 00" | 1412-1635 | 825 | 843 | 10.4 | 30.4 | 16.4 | 29.6 | 88.7 | 89.5 |
|  |  | Adami Tullu | 17 | 07° 51' 34" | 38° 41' 00" | 1367-1650 | 743 | 810 | 12.5 | 29.5 | 14.2 | 27.3 | 92.5 | 90.1 |
|  | West Arsi | Arsi Negelle | 18 | 07° 19' 60" | 38° 40' 00" | 1156-1454 | 780 | 767 | 11.6 | 25.0 | 15.0 | 25.5 | 76.1 | 80.2 |
|  |  | Seraro | 16 | 07° 18′ 45" | 38° 16′ 60" | 1234-1500 | 667 | 675 | 18.2 | 25.3 | 16.2 | 26.7 | 82.6 | 80.7 |
|  | West Harargae | Mieso | 19 | 08° 58′ 55" | 40° 23′ 60" | 920-1245 | 770 | 690 | 19.0 | 31.3 | 18.3 | 29.3 | 66.7 | 65.3 |
|  |  | Gemechis | 15 | 09° 05′ 00" | 41° 16′ 00" | 1300-1670 | 800 | 817 | 14.3 | 30.5 | 15.0 | 30.4 | 86.3 | 89.5 |
|  | East Harargae | Fedis | 12 | 08° 50′ 00" | 41° 59′ 55" | 908-1100 | 600 | 610 | 15.5 | 31.0 | 17.0 | 30.0 | 72.7 | 68.7 |
|  |  | Babile | 12 | 09° 08' 47" | 42° 13' 44" | 1287-1441 | 580 | 560 | 16.4 | 29.0 | 15.6 | 31.5 | 74.4 | 71.5 |
| Amhara | North Shewa | Merhabete | 14 | 08° 54' 00" | 39° 55' 00" | 1234-1567 | 875 | 930 | 13.5 | 26.0 | 14.5 | 25.6 | 77.3 | 79.6 |
|  |  | Minjarna Shenkora | 16 | 09° 38' 35" | 39° 19' 44" | 1421-1541 | 865 | 874 | 15.2 | 28.4 | 14.6 | 27.5 | 79.4 | 80.5 |
|  |  | Hageremariam K | 17 | 09° 19' 55" | 39° 14' 49" | 1213-1340 | 614 | 576 | 16.5 | 31.3 | 17.0 | 31.0 | 78.0 | 76.3 |
|  |  | Kewot | 12 | 10° 57' 50" | 38° 36' 00" | 1320-1431 | 913 | 982 | 14.5 | 27.7 | 14.0 | 28.0 | 71.2 | 75.1 |
|  | South Gondar | Lebo Kemkem | 8 | 12° 40' 50" | 37° 26' 40" | 1727-1830 | 750 | 885 | 16.5 | 33.0 | 17.0 | 32.5 | 88.0 | 93.5 |
|  |  | Tach Gayint | 14 | 11° 33' 50" | 38° 36' 00" | 1422 -1563 | 878 | 913 | 13.6 | 27.7 | 14.3 | 28.5 | 89.7 | 86.4 |
|  |  | Simada | 15 | 11° 24' 20" | 38° 23' 47" | 1260-1376 | 1200 | 1145 | 16.3 | 26.6 | 17.0 | 28.3 | 70.5 | 73.8 |
|  | South Wello | Kallu | 14 | 11° 53' 33" | 38° 48' 34" | 1411-1622 | 876 | 817 | 13.5 | 27.3 | 13.7 | 27.7 | 91.7 | 89.0 |
|  |  | Tehuluader | 13 | 11° 19' 40" | 39° 41' 34" | 890-1243 | 1007 | 976 | 17.0 | 27.0 | 16.3 | 26.4 | 67.4 | 68.7 |
| Central Ethiopia | Guragae | Abeshge | 18 | 08° 12' 19" | 37° 34' 29" | 1200-1347 | 896 | 837 | 14.3 | 30.0 | 15.0 | 29.6 | 67.5 | 71.5 |
|  |  | Sodo | 15 | 07° 16' 59" | 38° 00' 00" | 1720-1879 | 884 | 902 | 15.3 | 29.4 | 13.0 | 30.5 | 93.0 | 90.5 |
|  | Halaba | Kulfo-Halaba | 16 | 08° 19' 51" | 38° 47' 23" | 1656–1750 | 961 | 896 | 14.0 | 31.0 | 13.8 | 31.0 | 80.7 | 85.6 |
|  | Mareko | Mareko | 17 | 08° 00' 39" | 38° 31' 53" | 1706–1790 | 856 | 834 | 15.1 | 27.2 | 14.5 | 28.6 | 86.0 | 84.3 |
| South Ethiopia | Wolaita | Gindo Koisha | 15 | 06° 52' 40" | 37° 26′ 59" | 731-1319 | 1245 | 1213 | 18.4 | 30.4 | 19.0 | 30.7 | 68.5 | 65.3 |
|  |  | Humbo | 17 | 06° 48' 19" | 37° 45′ 15" | 1534-1712 | 1220 | 1143 | 18.0 | 24.0 | 15.5 | 27.7 | 84.7 | 85.9 |
|  | Gofa | Bonke | 18 | 06° 24' 60" | 36° 54' 60" | 870-1280 | 941 | 1130 | 12.5 | 28.0 | 11.0 | 27.6 | 69.0 | 72.9 |
|  |  | Geze-Gofa | 20 | 06° 14' 60" | 37° 00' 00" | 1461-1619 | 1401 | 1365 | 12.7 | 28.4 | 14.0 | 27.2 | 77.7 | 81.3 |
|  | Gamo | Arbaminch-zuria | 18 | 06° 04' 55'' | 37° 34' 00'' | 1186-1465 | 855 | 868 | 14.3 | 27.0 | 13.7 | 28.0 | 71.4 | 74.3 |
|  |  | Deramalo | 16 | 06° 14' 55'' | 37° 34' 00'' | 1354-1700 | 1156 | 1197 | 13.5 | 26.7 | 14.1 | 27.2 | 88.1 | 88.9 |
|  | Ari | Jinka (South Ari) | 14 | 07° 19' 60" | 38° 40' 00" | 1440-1558 | 1112 | 1213 | 15.7 | 30.3 | 16.1 | 31.0 | 80.7 | 82.7 |
|  | Basketo | Basketo SD | 12 | 06° 15' 00" | 36° 35' 00" | 770-1320 | 1236 | 1234 | 13.6 | 28.0 | 14.3 | 27.6 | 67.4 | 69.7 |
| South West Ethiopia | Keffa | Bita | 12 | 07° 14' 41" | 35° 41' 00" | 1000-1305 | 1541 | 1534 | 12.6 | 24.7 | 13.0 | 25.1 | 70.7 | 67.4 |
|  |  | Chena | 13 | 07° 15' 41" | 35° 46' 00" | 1000-1265 | 1622 | 1643 | 12.6 | 24.7 | 13.0 | 25.1 | 64.5 | 65.7 |
|  | Bench Shako | Debub Bench | 12 | 06° 55' 00" | 35° 10' 00" | 1312-1457 | 1627 | 1631 | 13.3 | 26.2 | 12.7 | 27.0 | 77.0 | 74.2 |
|  |  | Shako | 13 | 06° 50' 00" | 34° 56' 57" | 980-1384 | 1501 | 1492 | 14.5 | 28.4 | 15.2 | 29.3 | 70.7 | 74.6 |

^*^ Potential mungbean-growing regions of Ethiopia, included in the study. Mean annual rainfall (mRF) of the main cropping season, temperature and relative humidity (RH) of each district were obtained from nearby meteorological stations. Altitudinal ranges of the inspected fields in each district were recorded by GPS, Mareko SD (special district); Basketo SD (special district); Hageremariam K. = Hageremariam Kesem
