# Supplementary material for: Distribution and Occurrence of Cercospora Leaf Spot of Mungbean (Vigna radiata) and Associated Agronomic Factors in Ethiopia
Source: Plant Environ Interact. 2026 Jul 24;7(4):e70194. doi: 10.1002/pei3.70194 (PMC13400991; doi:10.1002/pei3.70194)
Supplement: Supplementary file 2 — Table S2: Outcomes of logistic regression analysis of the likelihood ratio test for predictor variables and variable categories with respect to CLS severity in mungbean‐growing areas of Ethiopia, during the 2024 and 2025 cropping seasons. [file PEI3-7-e70194-s002.docx]

**Table S2** | Outcomes of logistic regression analysis of the likelihood ratio test for predictor variables and variable categories with respect to CLS severity in mungbean-growing areas of Ethiopia, during the 2024 and 2025 cropping seasons

| **Entered variable** | **Likelihood ratio test for CLS severity** | | | | | | | |
| --- | --- | --- | --- | --- | --- | --- | --- | --- |
|  | **Residual D** | **df** | **D↓** | ***p* > χ ^2^** | **Variable category** | **ME** | **SE** | **ORs** |
| Intercept | 9207.0 | - | - | - | Intercept | -0.975 | 0.7967 | 0.58 |
| District | 2309.1 | 32.0 | 7148.5 | <0.0001 | Awash Melkass | 0.866 | 0.0261 | 2.90 |
|  |  |  |  |  | Adami Tullu | 0.922 | 0.0193 | 3.07 |
|  |  |  |  |  | Arsi Negelle | 0.578 | 0.0810 | 1.85 |
|  |  |  |  |  | Seraro | 0.599 | 0.0447 | 2.37 |
|  |  |  |  |  | Mieso | 0* | - | 1.00 |
|  |  |  |  |  | Gemechis | 0.743 | 0.0433 | 2.53 |
|  |  |  |  |  | Fedis | -0.572 | 0.1117 | 1.19 |
|  |  |  |  |  | Babile | 0.247 | 0.8473 | 1.98 |
|  |  |  |  |  | Merhabete | 0.729 | 0.0904 | 2.04 |
|  |  |  |  |  | Minjarna Shenkora | 0.571 | 0.0907 | 2.08 |
|  |  |  |  |  | Hageremariam K | -0.388 | 0.0942 | 1.68 |
|  |  |  |  |  | Kewot | 0.572 | 0.0762 | 1.98 |
|  |  |  |  |  | Lebo Kemkem | 0.812 | 0.0465 | 2.97 |
|  |  |  |  |  | Tach Gayint | 0.744 | 0.0347 | 2.71 |
|  |  |  |  |  | Simada | 0.580 | 0.0785 | 2.22 |
|  |  |  |  |  | Kallu | 0.746 | 0.0458 | 3.05 |
|  |  |  |  |  | Tehuluader | -0.881 | 0.1073 | 1.10 |
|  |  |  |  |  | Abeshge | 0.577 | 0.0828 | 2.11 |
|  |  |  |  |  | Sodo | 1.053 | 0.0165 | 3.17 |
|  |  |  |  |  | Kulfo-Halaba | 0.819 | 0.0447 | 2.67 |
|  |  |  |  |  | Mareko SD | 0.815 | 0.0627 | 2.79 |
|  |  |  |  |  | Gindo Koisha | -1.08 | 0.1290 | 0.95 |
|  |  |  |  |  | Humbo | 0.749 | 0.0645 | 2.51 |
|  |  |  |  |  | Bonke | -0.582 | 0.1152 | 1.32 |
|  |  |  |  |  | Geze-Gofa | -0.356 | 0.0726 | 1.90 |
|  |  |  |  |  | Arbaminch-zuria | -0.402 | 0.0991 | 1.58 |
|  |  |  |  |  | Daramalo | 0.757 | 0.0618 | 2.38 |
|  |  |  |  |  | Jinka | -0.567 | 0.0919 | 1.70 |
|  |  |  |  |  | Basketo SD | -0.503 | 0.1073 | 1.19 |
|  |  |  |  |  | Bita | -0.515 | 0.1144 | 1.19 |
|  |  |  |  |  | Chena | -0.596 | 0.1181 | 1.10 |
|  |  |  |  |  | Debub Bench | -0.453 | 0.0966 | 1.58 |
|  |  |  |  |  | Shako | -0.560 | 0.1164 | 1.10 |
| Year | 1107.4 | 1 | 6399.6 |  | 2024 | -0.079 | 0.0925 | 0.88 |
|  |  |  |  |  | 2025 | 0* | - | 1.00 |
| Altitude | 1198.1 | 2 | 5311.3 |  | <1000 | -0.091 | 0.1130 | 0.73 |
|  |  |  |  |  | 1000-1500 | 0* | - | 1.00 |
|  |  |  |  |  | >1500 | 0.899 | 0.0096 | 1.30 |
| Cropping system | 677.6 | 2 | 5879.5 |  | Sole cropping | 0* | - | 1.00 |
|  |  |  |  |  | Mixed cropping | 0.921 | 0.0075 | 1.31 |
|  |  |  |  |  | Intercropping | -0.137 | 0.1121 | 0.50 |
| Plant density | 858.3 | 1 | 5174.8 | <0.0001 | <40m^-2^ | 0* | - | 0.53 |
|  |  |  |  |  | >40m^-2^ | 0. 967 | 0.0108 | 1.00 |
| Plant growth stage | 514.7 | 2 | 4106.4 | <0.0001 | Vegetative | -0.060 | 0.1205 | 0.34 |
|  |  |  |  |  | Flowering | 0.522 | 0.0125 | 0.79 |
|  |  |  |  |  | Grain-filling | 0* | - | 1.00 |

**Table S2** | Continued

| **Entered variable** | **Likelihood ratio test for CLS severity** | | | | | | | |
| --- | --- | --- | --- | --- | --- | --- | --- | --- |
|  | **Residual D** | **df** | **D↓** | ***p* > χ ^2^** | **V. category** | **ME** | **SE** | **ORs** |
| Weed infestation level/status | 446.3 | 3 | 4798.8 | <0.0001 | none | -0.126 | 0.1039 | 0.70 |
|  |  |  |  |  | Low | 0* | - | 1.00 |
|  |  |  |  |  | Moderate | 0.842 | 0.0114 | 1.36 |
|  |  |  |  |  | High | 0.931 | 0.0078 | 1.55 |
| Weeding frequency | 378.6 | 2 | 5009.0 | <0.0001 | once | 0.950 | 0.0033 | 2.23 |
|  |  |  |  |  | twice | 0* | - | 1.00 |
|  |  |  |  |  | > 3 times | -0.108 | 0.0891 | 0.84 |
| Tillge | 402.5 | 2 | 4469.7 | <0.0001 | 2 times | 0.913 | 0.0086 | 1.48 |
|  |  |  |  |  | 3 times | 0* | - | 1.00 |
|  |  |  |  |  | 4 times | -0.085 | 0.1520 | 0.88 |
| Mungbean cultivar used | 289.2 | 1 | 3988.3 | <0.0001 | Improved | 0.843 | 0.0074 | 0.45 |
|  |  |  |  |  | Traditional | 0* | - | 1.00 |
| CI varieties | 391.0 | 2 | 4397.2 | <0.0001 | Rasa (N-26) | 0* | - | 1.00 |
|  |  |  |  |  | NVL-1 | -0.093 | 0.0350 | 0.73 |
|  |  |  |  |  | Shewarobit | 0.912 | 0.0059 | 1.63 |
| Seed Source | 221.5 | 2 | 3467.2 | <0.0001 | Agri-offices | -0.114 | 0.0990 | 0.43 |
|  |  |  |  |  | Local market | 0* | - | 1.00 |
|  |  |  |  |  | Farmers | 0.930 | 0.0048 | 0.61 |
| Sowing time | 360.4 | 2 | 4279.6 | <0.0001 | Mid-February | -0.124 | 0.0296 | 0.62 |
|  |  |  |  |  | Early April | -0.134 | - | 0.39 |
|  |  |  |  |  | Mid of July | 0* | - | 1.00 |
| Inorganic fertilizer | 141.8 | 1 | 1659.7 | <0.0001 | Applied | 0* | - | 1.00 |
|  |  |  |  |  | Not applied | -0.077 | 0.0248 | 2.06 |
| Waterlogging | 175.2 | 1 | 2001.5 | <0.0001 | Absent | 0* | - | 1.00 |
|  |  |  |  |  | Present | 0.990 | 0.0075 | 2.75 |

Residual D. = Residual deviance; df = degrees of freedom; D↓ = deviance reduction; *p* = probability of χ ^2^ value greater than the value of D↓; χ ^2^ = Chi-square; V. category = variable category; ME = coefficient of model estimate (stable model estimates); SE = standard error.
